# TRPV4-mediated calcium influx contributes temperature-dependent SARS-CoV-2 replication

**DOI:** 10.1371/journal.ppat.1014520

**Published:** 2026-08-21

**Authors:** Moe Kobayashi, Hatsumi Abe, Hikaruko Sugihara, Nene Kobayashi, Seira Omori, Yasuhiro Yamada, Takeshi Ichinohe

**Affiliations:** 1 Division of Viral Infection, Department of Infectious Disease Control, International Research Center for Infectious Diseases, Institute of Medical Science, The University of Tokyo, Tokyo, Japan; 2 Department of Molecular Pathology, Graduate School of Medicine and Faculty of Medicine, The University of Tokyo, Tokyo, Japan; 3 The University of Tokyo Pandemic Preparedness, Infection and Advanced Research Center (UTOPIA), The University of Tokyo, Tokyo, Japan; Duke-NUS Medical School, SINGAPORE

## Abstract

Temperature varies across the nasal cavity, lower respiratory tract, and during febrile conditions, yet the impact of these differences on SARS-CoV-2 replication remains poorly understood. Here, we show that ancestral SARS-CoV-2, Delta, or Omicron BA.5 variants replicate most efficiently at 37°C. We found that transient receptor potential vanilloid 4 (TRPV4)-mediated calcium influx, a thermosensitive cation channel known to be activated at 37°C, contributes to efficient SARS-CoV-2 replication. In addition, a calcineurin inhibitor, cyclosporine A (Cys A), and manidipine, an FDA-approved calcium channel blocker, both suppressed viral replication and protected Syrian hamsters from lethal infection with the SARS-CoV-2 Delta variant. Notably, a selective TRPV4 antagonist also conferred protection in infected hamsters. These results suggest that TRPV4-mediated calcium influx contributes to efficient SARS-CoV-2 replication at 37°C and highlight manidipine as a strong candidate for repurposing as an anti-SARS-CoV-2 therapeutic.

## Author summary

The SARS-CoV-2 that causes coronavirus disease 2019 (COVID-19), including its Delta and Omicron BA.5 variants, replicates efficiently at core body temperature of 37°C. Here we demonstrated that the thermo-sensitive cation channel TRPV4, which is activated at this temperature, plays a key role in the SARS-CoV-2 replication. Blocking the TRPV4 signals with certain drugs—like cyclosporine A or manidipine, a medication already approved for blood pressure treatment—reduced the SARS-CoV-2 replication in vitro and in vivo. These drugs also protected Syrian hamsters from lethal SARS-CoV-2 Delta variant infection. Our finding suggests that manidipine could be repurposed as a treatment for COVID-19, offering a potential new tool to fight the disease.

## Introduction

The emergence of SARS-CoV-2 and its subsequent variants has presented significant challenges to global public health. Despite the development of vaccines targeting the spike protein of the virus, the necessity for effective therapeutic interventions persists, particularly in the management of severe cases and the prevention of further spread until the achievement of widespread immunity. The Omicron variants, like other strains, have been observed to exhibit enhanced proliferation in the upper respiratory tract, a distinguishing feature from earlier variants that primarily affected the lower respiratory tract [1,2]. This difference in tissue tropism among SARS-CoV-2 variants is likely associated with variations in temperature sensitivity [3], as the upper respiratory tract tends to be cooler (approximately 33–35°C) compared to the core body temperature of 37°C.

The temperature-dependent replication efficiency of respiratory viruses, including rhinovirus, influenza virus, and SARS-CoV-2, represents a significant area of interest, particularly with regard to understanding the host innate and adaptive immune responses to viral infection [4–7]. A common symptom of both influenza and COVID-19 is a fever exceeding 38°C. Several studies have demonstrated that under such elevated temperatures, the replication of both the influenza virus and SARS-CoV-2 is suppressed in vitro and in vivo [3,6–8]. The host innate antiviral response to respiratory virus infection, including fever, gives rise to a dynamic environment in which the replication efficiency of the virus may be affected, depending on thermosensitive host factors [9–12].

One such factor that has gained attention is the TRPV4, a thermosensitive cation channel that has been demonstrated to be activated at physiological core body temperature of 37°C [13]. TRPV4 is involved in a number of physiological processes, including the regulation of calcium influx into cells [14]. Calcium signaling has been linked to the replication of several viruses, including influenza virus and SARS-CoV-2 [15–18], suggesting a potential role for TRPV4 in facilitating viral proliferation. Previous studies have demonstrated that the activation of TRPV4 channels can lead to an elevation in intracellular calcium levels, which subsequently facilitates the replication of certain viruses [19,20].

In this study, we examine the SARS-CoV-2 replication at 34°C, 37°C, or 39°C. We demonstrate that 37°C represents the optimal temperature for the replication of all examined SARS-CoV-2 variants. In addition, the TRPV4-mediated calcium influx appears to contribute to SARS-CoV-2 replication at 37°C. Finally, we demonstrate for the first time that manidipine, a calcium channel blocker, has significant effects in suppressing the severity of SARS-CoV-2 infection in Syrian hamsters. Notably, a selective TRPV4 antagonist also conferred protection in infected hamsters. These findings indicate manidipine as a strong candidate for repurposing as an anti-SARS-CoV-2 therapeutic, particularly in severe cases where viral replication needs to be controlled.

## Results

### Replication of SARS-CoV-2 variants at different temperatures

**Fig 1:**
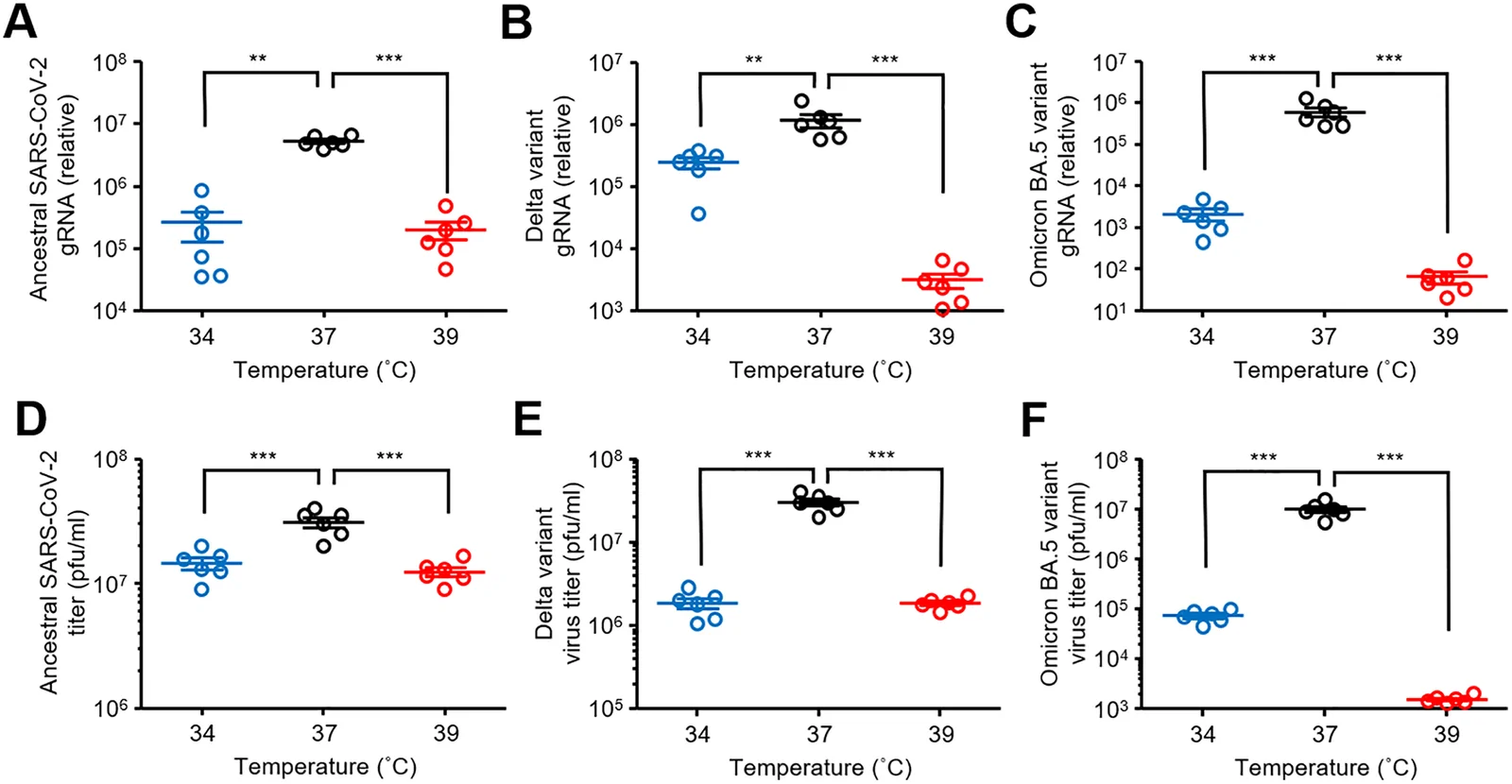
Replication of SARS-CoV-2 variants at different temperatures. **(A-F)** VeroE6/TMPRSS2 cells were infected with ancestral **(A and D)**, SARS-CoV-2 Delta **(B and D)**, or Omicron BA.5 variant (C amd F) at indicated temperatures. Total RNAs were extracted from cell-free supernatants at 24 (A, B) or 48 (C) h p.i. and SARS-CoV-2 N gRNA levels were assessed by quantitative reverse transcription PCR. Relative gRNA levels were normalized to the value obtained from the supernatants of mock-infected cells, which was set to 1 **(A-C)**. Cell-free supernatants were collected at 24 (D, E) or 48 (F) h p.i. and analyzed for virus titer by standard plaque assay **(D-F)**. Each symbol indicates individual values. Statistical significance was analyzed by two-way analysis of variance (ANOVA). **P < 0.01, ***P < 0.001. Values were normalized to mock controls within each independent experiment; therefore, absolute values are not intended for direct quantitative comparison across different figures.

SARS-CoV-2 Omicron variants have been observed to proliferate more efficiently in the human upper respiratory tract than in the lower respiratory tract [1,2]. The temperature within the nasal cavity is maintained at a range of 33–35°C, while the temperature within the lower respiratory tract is approximately 37°C. Infection with the influenza virus or SARS-CoV-2 is typically accompanied by the onset of a fever, reaching a body temperature of 38°C or above. Therefore, we sought to investigate how SARS-CoV-2 replicates at different temperatures. To this end, we infected VeroE6/TMPRSS2 cells with ancestral SARS-CoV-2, Delta, or Omicron variants and cultured for 24 or 48 hours at 34°C, 37°C, or 39°C. Importantly, culturing VeroE6/TMPRSS2 cells at these temperatures did not result in detectable cytotoxicity, as assessed by LDH release (S1 Fig). The ancestral SARS-CoV-2, Delta, and Omicron BA.5 variants exhibited enhanced replication efficiency at 37°C relative to 33°C or 40°C (Fig 1). To further characterize the dynamics of temperature-dependent viral replication, we performed a time-course analysis of SARS-CoV-2 replication at multiple time points post-infection. Consistent with our initial observations, viral replication was most efficient at 37°C across all tested variants, while replication was reduced at both 34°C and 39°C throughout the time course (S2 Fig).

To determine whether the observed temperature-dependent replication of SARS-CoV-2 is specific to VeroE6/TMPRSS2 cells, which lack type I interferon responses, we next examined viral replication in human respiratory cell models, including Calu-3 cells and primary human tracheal epithelial cells (HTEpC), both of which retain intact innate immune signaling pathways. Consistent with our observations in VeroE6/TMPRSS2 cells, ancestral SARS-CoV-2, Delta, and Omicron BA.5 variants replicated most efficiently at 37°C compared to 34°C or 39°C in both Calu-3 and HTEpC cells (S3 Fig). These results indicate that the temperature-dependent replication phenotype of SARS-CoV-2 is not restricted to interferon-deficient cell lines but is conserved in physiologically relevant human airway epithelial cells. Therefore, this phenomenon likely reflects a fundamental property of virus-host interactions rather than an artifact of the experimental system. For mechanistic studies, representative variants were selected to ensure experimental feasibility while capturing conserved features of temperature-dependent viral replication.

### Intrinsic RdRp activity does not account for temperature-dependent replication

To assess whether the intrinsic activity of the viral RNA-dependent RNA polymerase (RdRp) contributes to the temperature-dependent replication of SARS-CoV-2, we measured RdRp activity using a cell-free assay system. Unexpectedly, RdRp activity was highest at 34°C and was reduced at 37°C and 39°C (S4 Fig). Notably, this assay is typically performed at 37°C according to the manufacturer’s protocol, yet higher activity was observed at the lower temperature. These results indicate that the enhanced replication of SARS-CoV-2 at 37°C cannot be explained by increased intrinsic RdRp activity alone.

### Role of TRPV4 on SARS-CoV-2 replication

**Fig 2:**
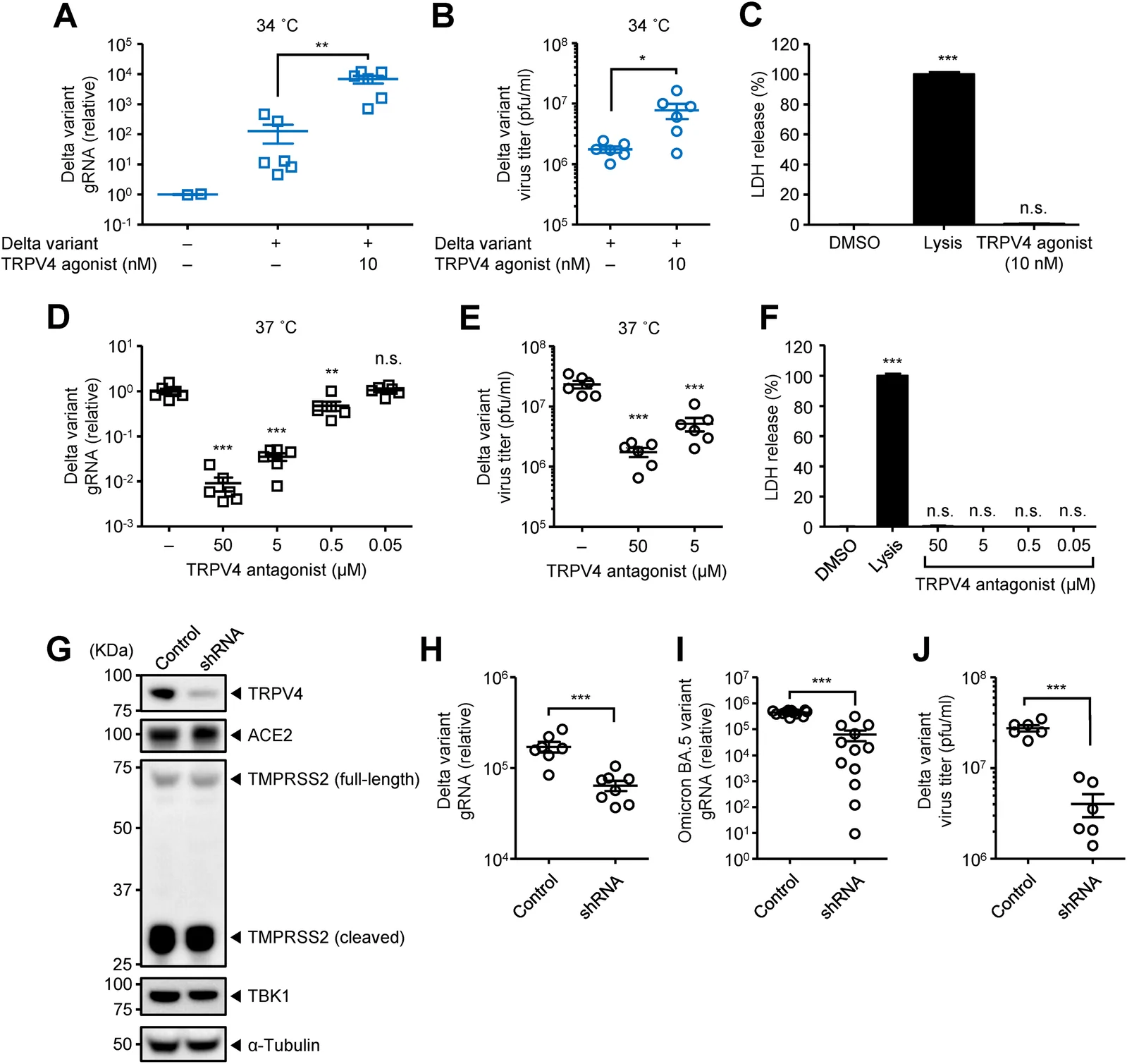
Role of TRPV4 on SARS-CoV-2 replication. **(A and B)** VeroE6/TMPRSS2 cells were infected with SARS-CoV-2 Delta variant in the presence or absence of TRPV4 agonist (10 nM) and cultured at 34°C. Total RNAs were extracted from cell-free supernatants at 24 h p.i. and SARS-CoV-2 N gRNA levels were assessed by quantitative reverse transcription PCR. Relative gRNA levels were normalized to the value obtained from the supernatants of mock-infected cells, which was set to 1 **(A)**. Cell-free supernatants were collected at 24 h p.i. and analyzed for virus titer by standard plaque assay **(B)**. **(C)** Uninfected VeroE6/TMPRSS2 cells were cultured in the presence or absence of TRPV4 agonist (10 nM) for 24 **h.** LDH activity was measured for cytotoxicity. **(D and E)** VeroE6/TMPRSS2 cells were infected with SARS-CoV-2 Delta variant in the presence or absence of TRPV4 antagonist and cultured at 37°C. Total RNAs were extracted from cell-free supernatants at 24 h p.i. and SARS-CoV-2 N gRNA levels were assessed by quantitative reverse transcription PCR. Relative gRNA levels were normalized to the value obtained from DMSO-treated infected control (–), which was set to 1 **(D)**. Cell-free supernatants were collected at 24 h p.i. and analyzed for virus titer by standard plaque assay **(E)**. **(F)** Uninfected VeroE6/TMPRSS2 cells were cultured with indicated amounts of TRPV4 antagonist for 24 **h.** LDH activity was measured for cytotoxicity. **(G)** Samples from VeroE6/TMPRSS2 cells stably expressing shRNA targeting TRPV4 mRNA were analyzed by western blotting using the indicated antibodies. **(H-J)** VeroE6/TMPRSS2 cells stably expressing shRNA targeting TRPV4 mRNA were infected with SARS-CoV-2 Delta (H and J) or Omicron BA.5 variant (I) at 37°C. Total RNAs were extracted from cell-free supernatants at 24 (H) or 48 (I) h p.i. and SARS-CoV-2 N gRNA levels were assessed by quantitative reverse transcription PCR. Relative gRNA levels were normalized to the value obtained from the supernatants of mock-infected cells, which was set to 1 (H and **I)**. Cell-free supernatants were collected at 24 h p.i. and analyzed for virus titer by standard plaque assay **(J)**. Each symbol indicates individual values. Statistical significance was analyzed by two-tailed unpaired Student’s t test (A, B, H-J) or two-way analysis of variance (ANOVA) **(C-F)**. *P < 0.05, **P < 0.01, ***P < 0.001, n.s., not significant. Values were normalized to mock controls within each independent experiment; therefore, absolute values are not intended for direct quantitative comparison across different figures.

Thermosensitive ion channels, such as TRPV4, play a crucial role in cellular responses to temperature fluctuations and have been implicated in various physiological and pathological processes, including viral infections. TRPV4 is activated within a temperature range close to human core body temperature [13], suggesting a potential role in mediating cellular responses at 37°C. Given its activation at physiological temperatures and its established role in calcium influx, TRPV4 was selected as a primary candidate to investigate the link between temperature and SARS-CoV-2 replication. We therefore hypothesized that TRPV4 might influence the replication efficiency of SARS-CoV-2 under different temperature conditions. To gain mechanistic insight into how core body temperature (37°C) supports SARS-CoV-2 replication, we first examined whether TRPV4 is involved in the temperature-dependent viral replication. To this end, we infected VeroE6/TMPRSS2 cells with a SARS-CoV-2 Delta variant in the presence or absence of GSK1016790A, a selective agonist for the TRPV4 receptor. Remarkably, treatment of VeroE6/TMPRSS2 cells with the TRPV4 agonist GSK1016790A significantly increased SARS-CoV-2 Delta variant replication at 34°C (Fig 2A and 2B), without affecting cytotoxicity (Fig 2C). In contrast, the same treatment did not enhance viral replication at 37°C or 39°C (S5 Fig), suggesting a temperature-dependent effect of TRPV4 activation. Conversely, treatment of VeroE6/TMPRSS2 cells with a selective TRPV4 channel antagonist (HC-067047) significantly suppressed SARS-CoV-2 Delta variant replication at 37°C (Fig 2D and 2E), without affecting cytotoxicity (Fig 2F). However, this antiviral effect was less pronounced at 34°C or 39°C, where only minimal or no significant inhibition was observed (S6 Fig). As these experiments were performed independently from the temperature-dependent replication assays (Fig 1), the magnitude of inhibition across different conditions should be interpreted with caution. To investigate the role of TRPV4 in SARS-CoV-2 replication, we generated VeroE6/TMPRSS2 cells stably expressing shRNA targeting TRPV4 mRNA. Western blot analysis confirmed the effective knockdown of TRPV4 protein without affecting the expression levels of ACE2, TMPRSS2, or TBK1 (Fig 2G). Notably, knockdown of TRPV4 significantly reduced the replication of both SARS-CoV-2 Delta and Omicron BA.5 variants, as evidenced by decreased levels of viral genomic RNA (gRNA) (Fig 2H and 2I) and infectious titers (Fig 2J). To further refine this interpretation, we generated a cold-adapted SARS-CoV-2 Omicron BA.5 variant that efficiently replicates at lower temperatures. As expected, the cold-adapted virus showed enhanced replication at 21°C compared to the parental virus (S7A Fig). Notably, under these low-temperature conditions, knockdown of TRPV4 did not significantly affect viral replication, as evidenced by comparable levels of viral gRNA and infectious titers between control and TRPV4 knockdown cells (S7B and S7C Fig). These results suggest that TRPV4 is not universally required for SARS-CoV-2 replication, but rather contributes to viral replication in a temperature-dependent manner.

### Role of calcium influx on SARS-CoV-2 replication

**Fig 3:**
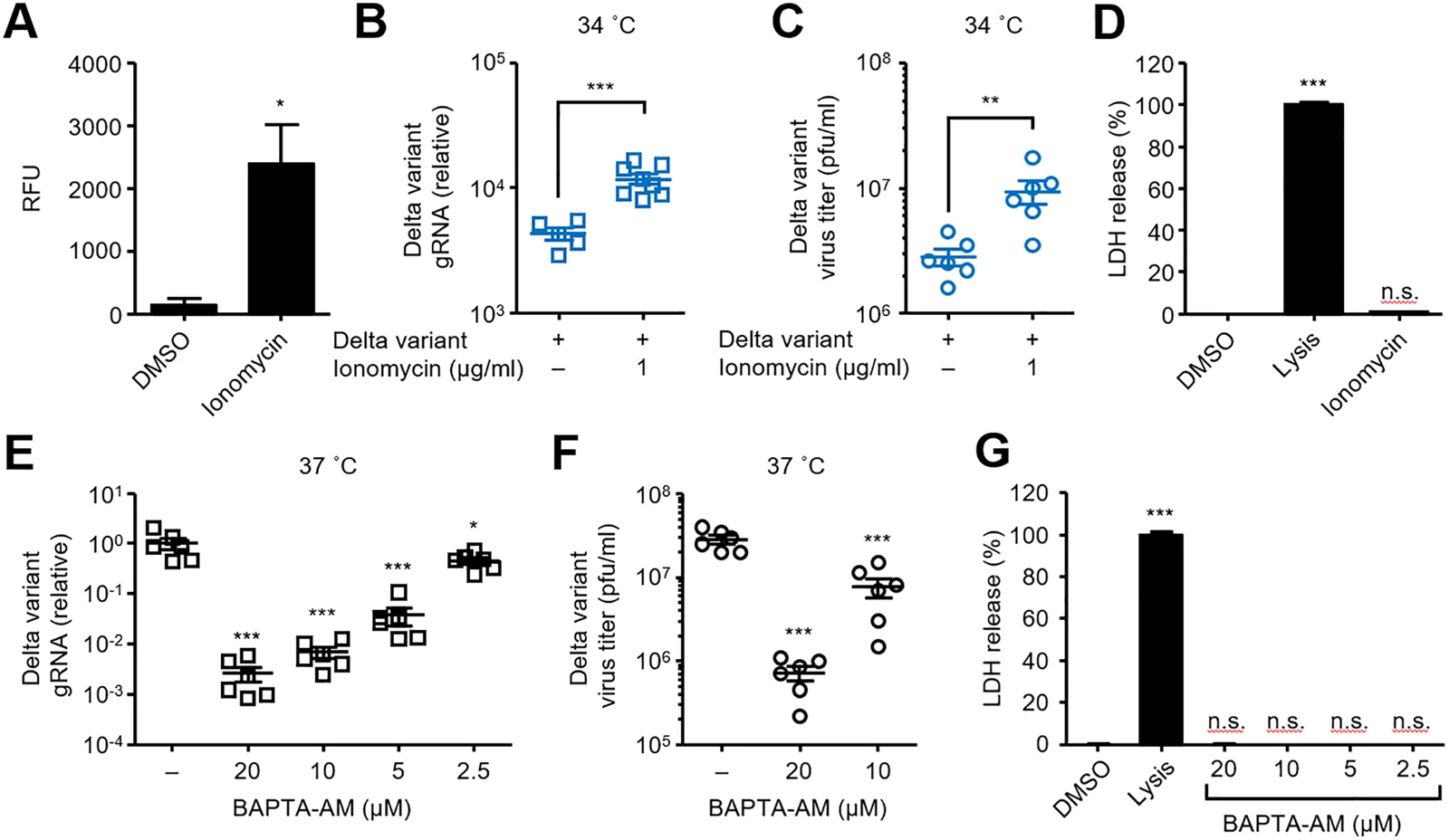
Role of calcium influx on SARS-CoV-2 replication. **(A)** Fluo-8 AM-treated VeroE6/TMPRSS2 cells were stimulated with ionomycin (1 μg/ml) for 5 min. Relative fluorescence units (RFU) were measured at 5 min after stimulation. **(B and C)** VeroE6/TMPRSS2 cells were infected with SARS-CoV-2 Delta variant in the presence or absence of ionomycin and cultured at 34°C. Total RNAs were extracted from cell-free supernatants at 24 h p.i. and SARS-CoV-2 N gRNA levels were assessed by quantitative reverse transcription PCR. Relative gRNA levels were normalized to the value obtained from the supernatants of mock-infected cells, which was set to 1 **(B)**. Cell-free supernatants were collected at 24 h p.i. and analyzed for virus titer by standard plaque assay **(C)**. **(D)** Uninfected VeroE6/TMPRSS2 cells were cultured in the presence or absence of ionomycin (1 μg/ml) for 24 **h.** LDH activity was measured for cytotoxicity. **(E and F)** VeroE6/TMPRSS2 cells were infected with SARS-CoV-2 Delta variant in the presence or absence of BAPTA-AM and cultured at 37°C. Total RNAs were extracted from cell-free supernatants at 24 h p.i. and SARS-CoV-2 N gRNA levels were assessed by quantitative reverse transcription PCR. Relative gRNA levels were normalized to the value obtained from DMSO-treated infected control (–), which was set to 1 **(D)**. Cell-free supernatants were collected at 24 h p.i. and analyzed for virus titer by standard plaque assay **(E)**. **(G)** Uninfected VeroE6/TMPRSS2 cells were cultured with indicated amounts of BAPTA-AM for 24 **h.** LDH activity was measured for cytotoxicity. Each symbol indicates individual values. Statistical significance was analyzed by two-tailed unpaired Student’s t test (A-C) or two-way analysis of variance (ANOVA) **(D-G)**. *P < 0.05, **P < 0.01, ***P < 0.001, n.s., not significant.

The activation of TRPV4 channels leads to calcium influx into cells [21,22]. Consistent with this, we confirmed that treatment of VeroE6/TMPRSS2 cells with a TRPV4 agonist resulted in a measurable calcium influx (S8 Fig). In addition, calcium influx in response to the TRPV4 agonist GSK1016790A, but not ATP, was significantly reduced in VeroE6/TMPRSS2 cells stably expressing shRNA targeting TRPV4 mRNA (S9 Fig). Furthermore, infection with SARS-CoV-2 variants induced calcium influx in VeroE6/TMPRSS2 cells (S10 Fig). In contrast, heat-inactivated SARS-CoV-2 failed to induce Ca^2+^ influx (S11 Fig), indicating that active viral processes, rather than mere viral particle binding, are required for the induction of calcium signaling. Notably, this SARS-CoV-2-induced Ca^2+^ influx was significantly attenuated in TRPV4 knockdown cells (S12 Fig), indicating that TRPV4 is a key mediator of virus-induced calcium entry. We therefore examined whether the enhanced SARS-CoV-2 replication by GSK1016790A treatment could be reproduced by treating cells with ionomycin, a selective calcium ionophore, at a lower temperature of 34°C. We first confirmed that treatment of VeroE6/TMPRSS2 cells with ionomycin resulted in a measurable calcium influx (Fig 3A). Indeed, treatment of VeroE6/TMPRSS2 cells with ionomycin resulted in a significant increase in SARS-CoV-2 Delta variant replication at 34°C (Fig 3B and 3C), without affecting cytotoxicity (Fig 3D). In contrast, the same treatment did not enhance viral replication at 37°C or 39°C (S13 Fig), indicating that ionomycin promotes viral replication specifically at 34°C but not at higher temperatures. Conversely, treatment of cells with the cell-permeable calcium chelator BAPTA-AM significantly suppressed the SARS-CoV-2 Delta variant replication at 37°C (Fig 3E and 3F), without affecting cytotoxicity (Fig 3G). However, no significant antiviral effect was observed at 34°C or 39°C following BAPTA-AM treatment (S14 Fig). Although ionomycin and BAPTA-AM broadly modulate intracellular Ca^2+^ levels and are not specific to TRPV4, these findings collectively suggest the importance of TRPV4-mediated calcium influx in the temperature-dependent replication efficiency of SARS-CoV-2.

### Calcineurin inhibitors suppress SARS-CoV-2 replication

**Fig 4:**
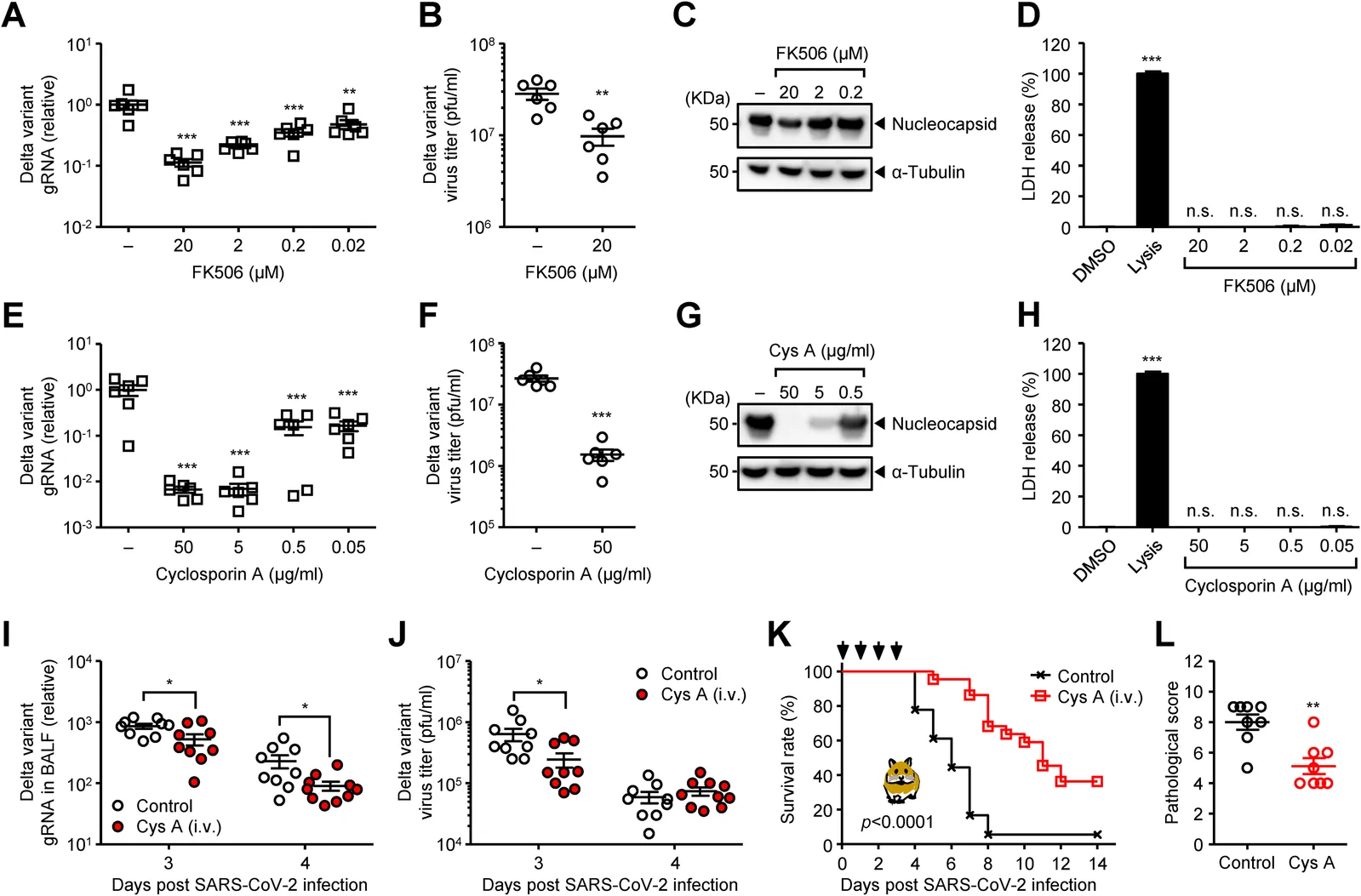
Calcineurin inhibitors suppress SARS-CoV-2 replication. **(A-C)** VeroE6/TMPRSS2 cells were infected with SARS-CoV-2 Delta variant in the presence or absence of FK506 and cultured at 37°C. Total RNAs were extracted from cell-free supernatants at 24 h p.i. and SARS-CoV-2 N gRNA levels were assessed by quantitative reverse transcription PCR. Relative gRNA levels were normalized to the value obtained from DMSO-treated infected control (–), which was set to 1 **(A)**. Cell-free supernatants were collected at 24 h p.i. and analyzed for virus titer by standard plaque assay **(B)**. Cell lysates were collected at 24 h p.i. and analyzed by immunoblotting with indicated antibodies **(C)**. **(D)** Uninfected VeroE6/TMPRSS2 cells were cultured with indicated amounts of FK506 for 24 **h.** LDH activity was measured for cytotoxicity. **(E-G)** VeroE6/TMPRSS2 cells were infected with SARS-CoV-2 Delta variant in the presence or absence of cyclosporine A (Cys A) and cultured at 37°C. Total RNAs were extracted from cell-free supernatants at 24 h p.i. and SARS-CoV-2 N gRNA levels were assessed by quantitative reverse transcription PCR. Relative gRNA levels were normalized to the value obtained from DMSO-treated infected control (–), which was set to 1 **(E)**. Cell-free supernatants were collected at 24 h p.i. and analyzed for virus titer by standard plaque assay **(F)**. Cell lysates were collected at 24 h p.i. and analyzed by immunoblotting with indicated antibodies **(G)**. **(H)** Uninfected VeroE6/TMPRSS2 cells were cultured with indicated amounts of Cys A for 24 **h.** LDH activity was measured for cytotoxicity. **(I-L)** Four-week-old Syrian hamsters infected with 8 × 10^6^ pfu of SARS-CoV-2 Delta variant were administered intravenously with PBS or Cys A (60 μg) at 0, 1, 2, and 3 days p.i. (arrow). Total RNAs were extracted from lung washes at indicated time points and SARS-CoV-2 N gRNA levels were assessed by quantitative reverse transcription PCR. Relative gRNA levels were normalized to the value obtained from the lung washes of mock-infected hamsters, which was set to 1 **(I)**. The lung washes were collected at indicated time points and viral titers were determined by standard plaque assay **(J)**. Mortality was monitored for 14 days **(K)**. Pathological scores were assessed based on histological examination of lung tissues collected at 5 days post SARS-CoV-2 infection, as described in S15 Fig **(L)**. Each symbol indicates individual values. Statistical significance was analyzed by two-way analysis of variance (ANOVA) (A, D, E, and **H)**, two-tailed unpaired Student’s t test (B, F, I, J, and **L)**, or two-sided log-rank (Mantel-Cox) test **(K)**. *P < 0.05, **P < 0.01, ***P < 0.001, n.s., not significant.

The precise reasons why the SARS-CoV-2 variants can replicate more efficiently at 37°C remain largely unclear. Given that the TRPV4 is activated at approximately 37°C [13], one potential explanation for the efficient replication of SARS-CoV-2 at 37°C is the TRPV4-mediated influx of calcium ions into the cell. Based on our previous data suggesting that elevation of intracellular calcium concentration promotes viral replication, we next investigated the role of calcineurin, a calcium-dependent protein phosphatase involved in intracellular signaling, in SARS-CoV-2 replication. To this end, we infected VeroE6/TMPRSS2 cells with a SARS-CoV-2 Delta variant in the presence or absence of calcineurin inhibitors, FK506 or cyclosporine A (Cys A). Treatment of VeroE6/TMPRSS2 cells with the FK506 or Cys A significantly suppressed SARS-CoV-2 replication at 37°C without affecting cytotoxicity (Fig 4A-4H). Furthermore, Cys A-treated hamsters showed significantly reduced viral loads at 3 days post-infection and improved survival compared to the control group, along with markedly lower pathological scores indicative of reduced lung inflammation (Fig 4I–4L).

### Manidipine protects hamsters from lethal SARS-CoV-2 infection

**Fig 5:**
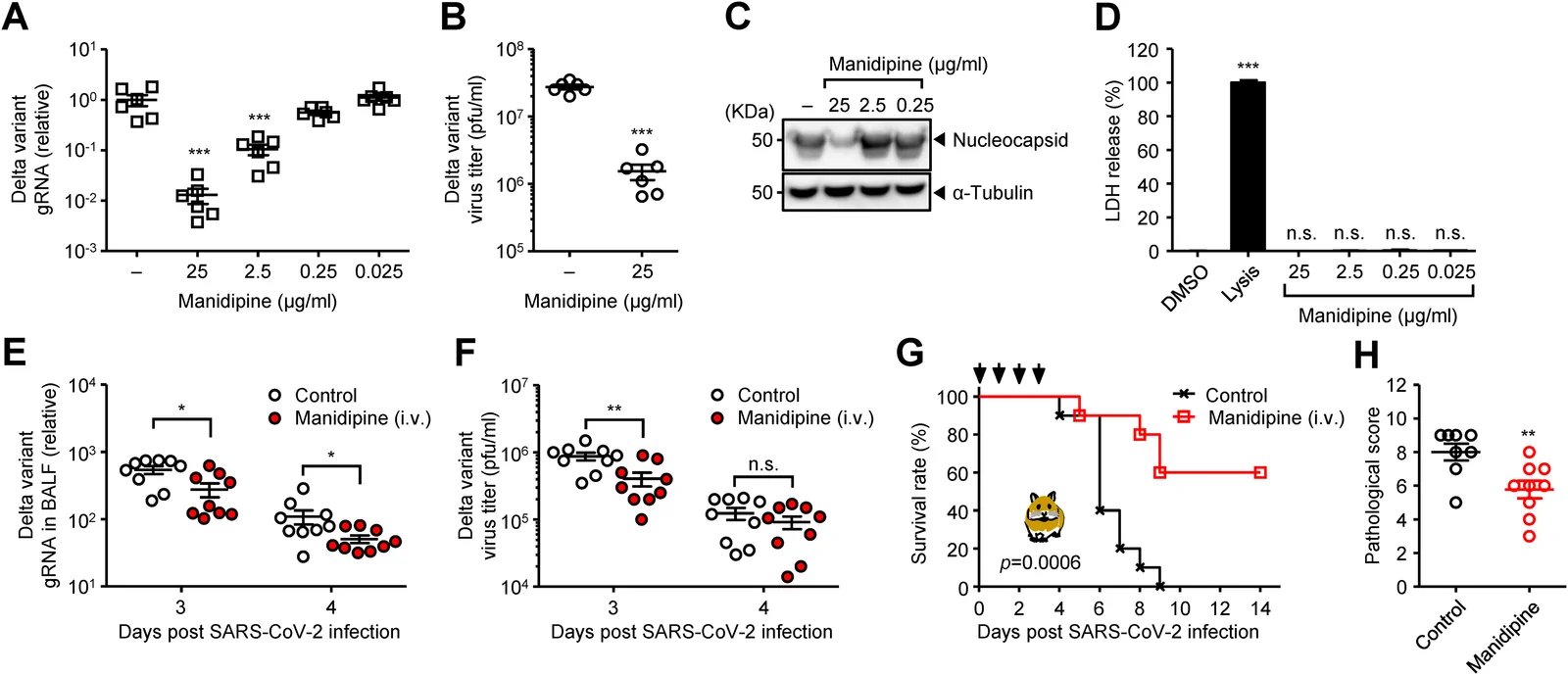
Manidipine protects hamsters from lethal SARS-CoV-2 infection. **(A-C)** VeroE6/TMPRSS2 cells were infected with SARS-CoV-2 Delta variant in the presence or absence of manidipine and cultured at 37°C. Total RNAs were extracted from cell-free supernatants at 24 h p.i. and SARS-CoV-2 N gRNA levels were assessed by quantitative reverse transcription PCR. Relative gRNA levels were normalized to the value obtained from DMSO-treated infected control (–), which was set to 1 **(A)**. Cell-free supernatants were collected at 24 h p.i. and analyzed for virus titer by standard plaque assay **(B)**. Cell lysates were collected at 24 h p.i. and analyzed by immunoblotting with indicated antibodies **(C)**. **(D)** Uninfected VeroE6/TMPRSS2 cells were cultured with indicated amounts of manidipine for 24 **h.** LDH activity was measured for cytotoxicity. **(E-H)** Four-week-old Syrian hamsters infected with 8 × 10^6^ pfu of SARS-CoV-2 Delta variant were administered intravenously with PBS or manidipine (15 μg) at 0, 1, 2, and 3 days p.i. (arrow). Total RNAs were extracted from lung washes at indicated time points and SARS-CoV-2 N gRNA levels were assessed by quantitative reverse transcription PCR. Relative gRNA levels were normalized to the value obtained from the lung washes of mock-infected hamsters, which was set to 1 **(E)**. The lung washes were collected at indicated time points and viral titers were determined by standard plaque assay **(F)**. Mortality was monitored for 14 days **(G)**. Pathological scores were assessed based on histological examination of lung tissues collected at 5 days post SARS-CoV-2 infection, as described in S15 Fig **(H)**. Each symbol indicates individual values. Statistical significance was analyzed by two-way analysis of variance (ANOVA) **(A and D)**, two-tailed unpaired Student’s t test (B, E, F, and **H)**, or two-sided log-rank (Mantel-Cox) test **(G)**. *P < 0.05, **P < 0.01, ***P < 0.001, n.s., not significant.

Manidipine is a calcium channel blocker that has been approved for use by the FDA. In vitro studies have demonstrated that manidipine inhibits the activity of the main protease (M^pro^) of SARS-CoV-2 and limits the viral replication [23–25]. However, it remains unclear whether manidipine is capable of suppressing SARS-CoV-2 replication in vivo and reducing the severity of the disease. First, we confirmed that treatment of VeroE6/TMPRSS2 cells with manidipine inhibited calcium influx induced by the TRPV4 agonist, ionomycin, and SARS-CoV-2 (Delta and BA.5 variants) (S15 Fig). Consistent with previous reports [23–25], treatment of VeroE6/TMPRSS2 cells with the manidipine significantly suppressed SARS-CoV-2 replication at 37°C (Fig 5A-5C), without affecting cytotoxicity (Fig 5D). To investigate the therapeutic efficacy of manidipine in vivo, we administered manidipine intravenously to Syrian hamsters following lethal SARS-CoV-2 Delta variant infection. Notably, the manidipine-treated hamsters showed significantly reduced viral loads at 3 days post-infection and improved survival compared to the control group, along with markedly lower pathological scores indicative of reduced lung inflammation (Fig 5E–5H). Our findings suggest that the TRPV4-mediated calcium influx contributes to efficient SARS-CoV-2 replication at physiological temperature, highlighting the potential of manidipine as an effective therapeutic agent against lethal SARS-CoV-2 infection in vivo.

### Manidipine protects hamsters from lethal SARS-CoV-2 infection

**Fig 6:**
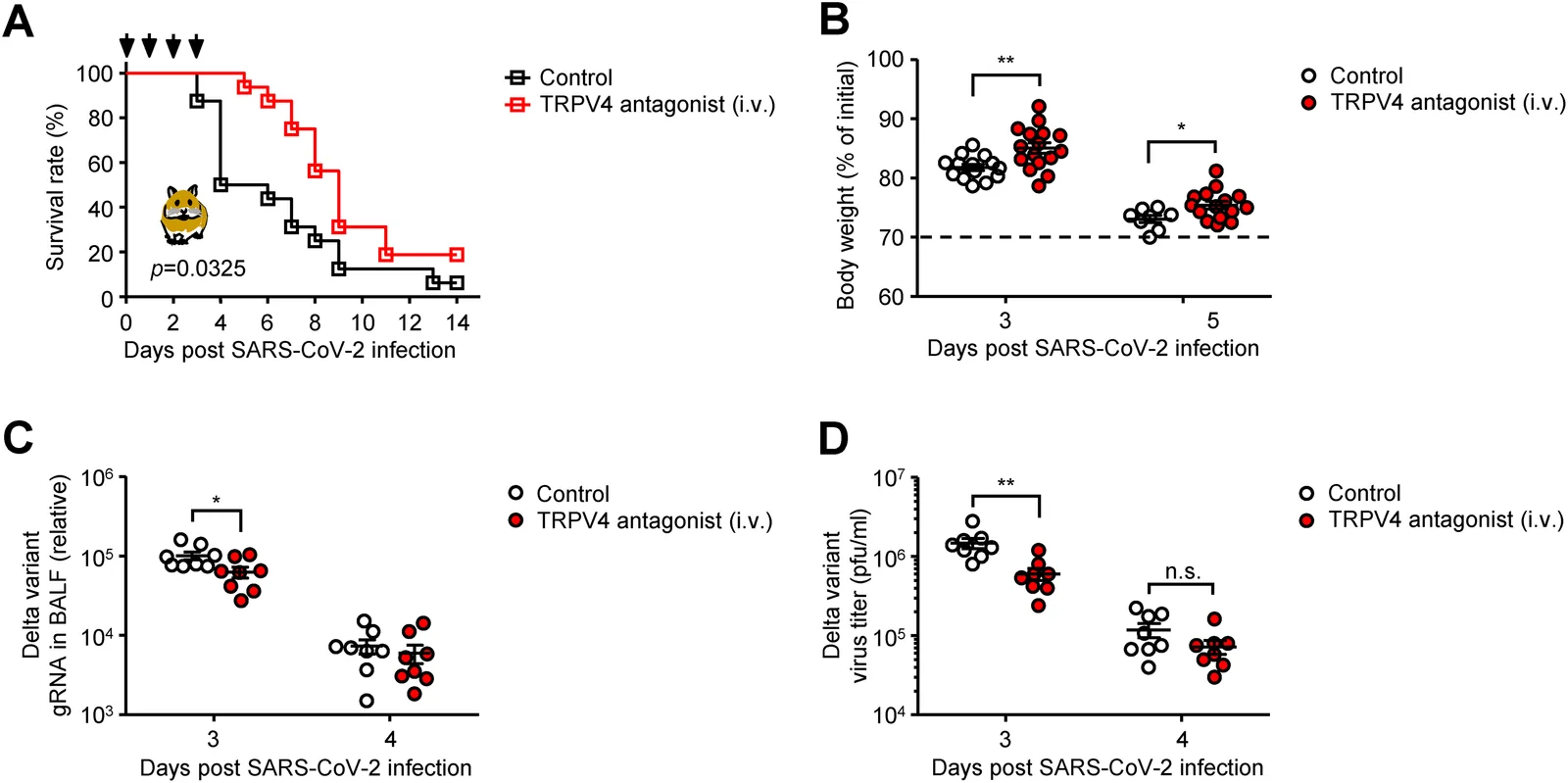
TRPV4 antagonist protects hamsters from lethal SARS-CoV-2 infection. **(A-D)** Four-week-old Syrian hamsters infected with 8 × 10^6^ pfu of SARS-CoV-2 Delta variant were administered intravenously with PBS or a TRPV4 antagonist (0.16 mg/kg) at 0, 1, 2, and 3 days p.i. (arrow). Mortality was monitored for 14 days **(A)**. Body weight changes following infection **(B)**. Total RNAs were extracted from lung washes at indicated time points and SARS-CoV-2 N gRNA levels were assessed by quantitative reverse transcription PCR. Relative gRNA levels were normalized to the value obtained from the lung washes of mock-infected hamsters, which was set to 1 **(C)**. The lung washes were collected at indicated time points and viral titers were determined by standard plaque assay **(D)**. Each symbol indicates individual values. Statistical significance was analyzed by two-sided log-rank (Mantel-Cox) test **(A)**, or two-tailed unpaired Student’s t test (B, C, and **D)**. *P < 0.05, **P < 0.01, n.s., not significant.

To further determine whether inhibition of TRPV4 signaling itself is sufficient to confer protection in vivo, independent of potential off-target effects of manidipine, we evaluated the therapeutic efficacy of a selective TRPV4 antagonist (HC-067047) in the Syrian hamster model of SARS-CoV-2 infection. Notably, intravenous administration of the TRPV4 antagonist significantly improved survival and attenuated body weight loss compared to control-treated animals (Fig 6A and 6B). In addition, viral gRNA levels in BALF were modestly reduced at early time points, although infectious virus titers were not significantly different at later time points (Fig 6C and 6D). These results demonstrate that pharmacological inhibition of TRPV4 signaling alone is sufficient to mitigate disease severity in vivo. Together with our in vitro findings, these results further support a model in which TRPV4-mediated calcium signaling contributes to SARS-CoV-2 pathogenesis in a temperature-dependent manner.

## Discussion

In this study, we examined the replication efficiency of SARS-CoV-2 variants at different temperatures and investigated the role of TRPV4-mediated calcium influx in the optimal replication of the virus at core body temperature (37°C). Our results demonstrated that 37°C is the optimal temperature for replication of all tested SARS-CoV-2 variants, including the ancestral, Delta, and Omicron variants, which showed enhanced proliferation at this temperature compared to lower (34°C) or higher (39°C) temperatures. Importantly, the temperature-dependent replication phenotype was consistently observed in human respiratory epithelial cells, indicating that this phenomenon is not dependent on the interferon-deficient nature of VeroE6/TMPRSS2 cells. Furthermore, we identified TRPV4-mediated calcium influx as an important contributor facilitating this temperature-dependent replication, suggesting that targeting TRPV4 may offer new therapeutic approaches for treating severe cases of SARS-CoV-2 infection. We also demonstrated for the first time that manidipine, a calcium channel blocker, significantly suppressed viral replication and improved survival in the SARS-CoV-2 Delta variant-infected hamsters, highlighting its potential as a repurposed antiviral therapy.

The findings of this study provide significant insights into the temperature-dependent replication dynamics of SARS-CoV-2 variants, highlighting a potential contribution of core body temperature in viral proliferation. The observation that 37°C represents the optimal temperature for the replication of SARS-CoV-2 variants is consistent with a previous report on the temperature sensitivity of SARS-CoV-2 [3,8]. Herder et al. show that elevated temperatures of 39°C or 40°C restricts ancestral SARS-CoV-2 replication in primary human bronchiolar epithelial cells relative to 37°C [8]. Following SARS-CoV-2 infection, the expression of innate antiviral genes was observed to be diminished in human epithelial bronchiolar cells cultured at 40°C relative to 37°C. Furthermore, even in Vero cells, which are known to be defective in type-I IFN responses, SARS-CoV-2 replication was inhibited at 40°C compared to 37°C. Therefore, the authors concluded that elevated temperature inhibits SARS-CoV-2 replication independently of IFN-mediated antiviral immune defenses [8]. To further examine whether intrinsic viral enzymatic activity contributes to temperature-dependent replication, we assessed SARS-CoV-2 RdRp activity in a cell-free system. Interestingly, RdRp activity was highest at 34°C, despite reduced viral replication at this temperature in cells. This discrepancy suggests that intrinsic viral enzymatic activity alone does not account for the enhanced replication at 37°C and instead highlights the importance of host-dependent mechanisms. Similarly, Kawaoka and colleagues show that higher temperature at 40°C suppresses the replication of SARS-CoV-2 Delta and Omicron BA.5 and BQ.1.1 variants in human alveolar epithelial cells and VeroE6/TMPRSS2 cells [3]. However, our study extends this understanding by identifying TRPV4-mediated calcium influx as an important host factor contributing to temperature-dependent SARS-CoV-2 replication. This finding suggests that viral replication at core body temperature may be influenced, at least in part, by host thermosensitive factors such as TRPV4 channels that regulate calcium entry into cells.

One of the important findings of this study is that TRPV4 contributes to efficient SARS-CoV-2 replication under physiological temperature conditions. Although our data support an important role for TRPV4-mediated calcium influx, they do not exclude the contribution of additional temperature-sensitive host factors. Previous studies have highlighted the importance of calcium signaling in the entry of influenza viruses and SARS-CoV-2 [15–18]. Recent reviews have further highlighted the importance of host cell calcium dynamics during SARS-CoV-2 infection and suggested that multiple calcium channels and signaling pathways contribute to viral replication and pathogenesis [26]. Notably, our data using heat-inactivated virus suggest that TRPV4 activation requires active viral processes rather than simple virion binding, implying that intracellular events associated with viral replication may contribute to TRPV4-mediated calcium influx. It is noteworthy that our findings regarding TRPV4 activation are in accordance with those of previous studies on other thermosensitive TRP channels, which have been demonstrated to regulate virus-host interactions [27,28]. While the respiratory syncytial virus (RSV) enhances the influx of calcium through TRPV1 channels [27], the calcium channel blocker Verapamil inhibits RSV infection by blocking calcium influx [29]. Furthermore, a TRPV1 antagonist inhibits chikungunya virus infection, whereas the activation of TRPV1 by resiniferatoxin, a TRPV1 agonist, significantly enhances the virus replication in macrophages [28]. The temperature-dependent activation of TRPV4 at 37°C may provide an explanation for the optimal replication of SARS-CoV-2 at core body temperature, as the influx of calcium ions can activate downstream signaling pathways that facilitate viral replication, such as calcineurin and NFAT (nuclear factor of activated T-cells) pathways [30–32]. In this context, calcineurin likely acts as a downstream effector of TRPV4-mediated Ca^2+^ signaling, linking temperature-dependent calcium influx to enhanced viral replication. This temperature specificity may reflect the fact that TRPV4 functions most effectively within a narrow physiological range close to core body temperature, whereas its contribution becomes limited at lower or higher temperatures. Outside this range, other host or viral factors may become dominant in determining replication efficiency. In this context, the TRPV4-dependent calcium influx at physiological temperature likely acts as a downstream effector of TRPV4-mediated Ca^2+^ signaling, linking temperature-dependent calcium influx to enhanced viral replication. Interestingly, our data using a cold-adapted SARS-CoV-2 variant demonstrate that TRPV4 is dispensable for viral replication at lower temperatures, suggesting that the contribution of TRPV4 is context-dependent and may be complemented by alternative pathways under non-physiological conditions. Although the present study did not identify the mutations responsible for cold adaptation, genome sequencing and functional characterization of the cold-adapted variant may provide valuable insights into the viral determinants of temperature-dependent replication and represent an important direction for future studies. The specificity of TRPV4 involvement is further supported by the selective impairment of TRPV4 agonist-induced Ca^2+^ influx, while ATP-induced responses remain intact. In addition to TRPV4-mediated calcium signaling, temperature may influence other critical steps in the viral life cycle, including receptor expression, viral entry efficiency, membrane fusion, and replication complex activity. Although these factors were not directly examined in the present study, no significant increase in LDH release was observed in VeroE6/TMPRSS2 cells cultured at 34°C, 37°C, or 39°C (S1 Fig), suggesting that the reduced viral replication observed at elevated temperature is unlikely to be attributable to overt temperature-induced cytotoxicity. Our data suggest that host-dependent mechanisms play a dominant role in temperature-dependent replication, and further investigation of these pathways will be important to fully understand the effects of temperature on SARS-CoV-2 infection. Therefore, understanding the mechanisms behind temperature-dependent replication will be crucial in developing more effective treatments against SARS-CoV-2 and its evolving variants.

The present study provides substantial evidence for the therapeutic potential of calcium channel blockers, particularly manidipine, in mitigating the replication and severity of SARS-CoV-2 Delta variant infection. Manidipine, a calcium channel blocker approved by the FDA, has previously been demonstrated to inhibit the M^pro^ of SARS-CoV-2 in vitro [23–25]. However, our results extend these findings by demonstrating that manidipine significantly suppresses SARS-CoV-2 Delta variant replication in Syrian hamsters. The reduction in viral load and improved survival observed in manidipine-treated hamsters provides compelling evidence that targeting calcium signaling could be a viable strategy for controlling viral replication in severe cases of COVID-19. The mechanism by which manidipine exerts its antiviral effects is likely related to its ability to inhibit calcium influx into host cells, thereby disrupting the calcium-dependent processes required for SARS-CoV-2 replication. Given that TRPV4-mediated calcium influx is critical for SARS-CoV-2 replication, it is plausible that manidipine interferes with this pathway, limiting viral proliferation. Importantly, our in vivo data using a TRPV4-specific antagonist further demonstrate that inhibition of TRPV4 signaling alone is sufficient to reduce disease severity and viral replication, supporting a host-targeted mechanism independent of direct viral Mpro inhibition. Furthermore, the suppression of calcineurin activity, a downstream effector of calcium signaling, by inhibitors such as FK506 and cyclosporine A provides additional support for the role of calcium-dependent pathways in SARS-CoV-2 replication [32]. Notably, the protective effect of cyclosporine A may reflect attenuation of host immunopathology rather than solely antiviral activity, consistent with our previous findings that excessive inflammatory responses, including TNF-α production, contribute to disease severity [33]. This may explain the apparent discrepancy between survival benefit and relatively modest reductions in viral load. These findings are consistent with previous reports on the antiviral effects of calcium channel blockers in other viral infections, including the influenza virus, severe fever with thrombocytopenia syndrome virus, dengue virus, hepatitis C virus, Zika virus, and Marburg virus [20,31,34–37]. The ability of manidipine to inhibit the calcium-dependent signaling pathways indicates that it may possess broad-spectrum antiviral activity and could be repurposed as a therapeutic option for the management of various virus-induced infectious diseases.

In conclusion, our study provides valuable insights into the temperature-dependent replication of SARS-CoV-2 and a role of TRPV4-mediated calcium influx in this process. Our data support TRPV4-mediated calcium influx as an important contributor to temperature-dependent SARS-CoV-2 replication. However, our findings do not exclude the contribution of additional temperature-sensitive host factors, and further studies will be required to define the complete molecular mechanism underlying temperature-dependent viral replication. The identification of manidipine as a potential therapeutic agent represents a significant advancement in the development of new treatments for SARS-CoV-2. Further research into the molecular mechanisms of calcium signaling in viral replication and the clinical application of calcium channel blockers could lead to substantial progress in the fight against SARS-CoV-2 and other viral pathogens.

## Materials and methods

### Animals

Four-week-old female Syrian hamsters were purchased from Japan SLC, Inc (Shizuoka, Japan).

### Cell culture

VeroE6 cells stably expressing transmembrane protease serine 2 (VeroE6/TMPRSS2; JCRB Cell Bank 1819) were maintained in Dulbecco’s modified Eagle’s medium (DMEM) (low-glucose) (Nacalai Tesque, 08456–65) supplemented with 10% v/v fetal bovine serum (FBS), 1% v/v penicillin (100 units/ml)/streptomycin (100 μg/ml), and G418 (1 mg/ml; Nacalai Tesque, 16512–94). Calu-3 (Cell Lines Service, 305032-ACADEMIC) cells were maintained in Eagle’s minimum essential medium (EMEM) (Cell Lines Service, 820100A) supplemented with 10% v/v FBS, 1% v/v penicillin (100 units/ml)/streptomycin (100 μg/ml), and 1% v/v non-essential amino acids (NEAA) (MP Biomedicals, IC1681049). Human tracheal epithelial cells (HTEpC) were cultured in Airway Epithelial Cell Growth Medium (PromoCell GmbH, C-21060). HaP-T1 cells (European Collection of cell cultures, EC93121054-F0) were cultured in DMEM (high-glucose) (Nacalai Tesque, 08458–16) supplemented with 10% v/v fetal bovine serum (FBS), 1% v/v penicillin (100 units/ml)/streptomycin (100 μg/ml), and 1% v/v NEAA.

### Viruses

An ancestral SARS-CoV-2 strain bearing aspartic acid at position 614 of spike (S) protein (S-614D) [38], the Delta variant hCoV-19/Japan/TY11–927-P1/2021 (lineage B.1.617.2, GISAID ID: EPI_ISL_2158617) [39], and the Omicron BA.5 variant hCoV-19/Japan/TY41–702/2022 (GISAID ID: EPI_ISL_13512581) [40] were grown in VeroE6/TMPRSS2 cells for 2 or 3 days at 37°C. Viral titers were quantified by a standard plaque assay using VeroE6/TMPRSS2 cells and viral stock was stored at -80°C.

For generation of a cold-adapted SARS-CoV-2 BA.5 variant, VeroE6/TMPRSS2 cells seeded in 6-well plates were infected with the SARS-CoV-2 BA.5 variant at a multiplicity of infection (MOI) of 1 for 1 h at 37°C. After infection, the cells were cultured in Dulbecco’s modified Eagle’s medium (DMEM) (low-glucose) supplemented with 5% v/v FBS, 1% v/v penicillin (100 units/ml)/streptomycin (100 μg/ml), and 1% v/v HEPES buffer (Nacalai Tesque, 17557–94) for an additional 3 days at 28°C in a CO_2_ incubator. This passaging procedure was repeated five times. The resulting virus was then inoculated onto fresh HaP-T1 cells seeded in 6-well plates and cultured in Leibovitz’s L-15 medium (Thermo Fisher Scientific, 11415064) supplemented with 5% v/v FBS, 1% v/v penicillin (100 units/ml)/streptomycin (100 μg/ml), and 1% v/v HEPES buffer (Nacalai Tesque, 17557–94) for an additional 3 or 4 days at 21°C in a non-CO_2_ incubator (Mitsubishi Electric Engineering, Cool Incubator CN-40A, 1-8963-01). This low-temperature passaging procedure was repeated ten times. The resulting virus was designated as the cold-adapted SARS-CoV-2 BA.5 variant, and stored at -80°C.

### SARS-CoV-2 infection

VeroE6/TMPRSS2 cells in 6-well plates were infected with SARS-CoV-2 at an MOI of 0.01 for 1 h at 37°C, and cultured with DMEM (low-glucose) supplemented with 5% FBS, 1% P/S, and 1% HEPES buffer (Nacalai Tesque, 17557–94) for an additional 23 h or 47 h at 34°C, 37°C, or 39°C.

For intranasal infection, hamsters were infected by intranasal application of 400 μL of virus suspension (8 × 10^6^ pfu of SARS-CoV-2 Delta variant) under isoflurane anaesthesia.

All experiments with SARS-CoV-2 were performed in enhanced biosafety level 3 (BSL3) containment laboratories at the University of Tokyo, in accordance with the institutional biosafety operating procedures. All animal experiments were performed in accordance with University of Tokyo’s Regulations for Animal Care and Use, which were approved by the Animal Experiment Committee of the Institute of Medical Science, the University of Tokyo (PA2233).

### Reagents

A selective agonist for the TRPV4 receptor (GSK1016790A, 530533) and a selective TRPV4 channel antagonist (HC-067047, 616521) were purchased from Merck Millipore. Ionomycin (19444–91) and BAPTA-AM (03731–24) were obtained from Nacalai Tesque. Calcineurin inhibitors, FK506 (10007965) and Cys A (AG-CN2–0079-M100), were purchased from Cayman Chemical Company and AdipoGen, Inc., respectively. Manidipine dihydrochloride was obtained from LKT Laboratories, Inc. (M0248).

### Quantitative PCR

Total RNA was extracted from cell-free supernatants or lung washes using TRIzol reagent (Invitrogen, 15596018) and reverse transcribed into cDNA using SuperScript III reverse transcriptase (Invitrogen, 18080085) with a SARS-CoV-2 N reverse primer (5’- tctggttactgccagttgaatctg-3’). TB Green Premix Ex Taq II (TaKaRa, RR820A) and a LightCycler 1.5 instrument (Roche Diagnostics) were used for quantitative PCR with the following primers: SARS-CoV-2 N forward, 5’- gaccccaaaatcagcgaaat-3’, and reverse, 5’- tctggttactgccagttgaatctg-3’. Relative viral gRNA levels were normalized to the value obtained from the supernatants of mock-infected cells, which was set to 1. As these values represent relative measurements normalized independently in each experiment, they should not be interpreted as absolute viral RNA copy numbers or directly compared across different experiments, figures, or with infectious virus titers determined by plaque assay.

### Western blot analysis

The virus-infected VeroE6/TMPRSS2 cells in 6-well plates were washed with PBS and lysed in 500 μl of 1 × TNT buffer (50 mM Tris [pH 7.5], 150 mM NaCl, 1% Triton X-100, 1 mM EDTA, 10% glycerol). Lysates were centrifuged at 20,630 × g for 10 min at 4°C. The supernatant was mixed with 4 × lithium dodecyl sulfate (LDS) sample buffer (Invitrogen, NP0007) and 10 × sample reducing agent (Invitrogen, NP0009). Samples were boiled for 5 min and fractionated by NuPAGE 10% Bis-Tris Protein Gels (Invitrogen, NP0316BOX) and electroblotted onto polyvinylidene difluoride (PVDF) membranes (Bio-Rad Laboratories, 170–4156). The membranes were incubated with mouse anti-α tubulin (Santa Cruz, sc-32293; 1:2000), rabbit anti-SARS-CoV-2 nucleocapsid (Cell Signaling, 33336; 1:1000), rabbit anti-TRPV4 (Invitrogen, PA5–41066; 1:1000), rabbit anti-ACE2 (abcam, ab108252; 1:1000), rabbit anti-TMPRSS2 (proteintech, 14437–1-AP; 1:1000), or mouse-anti-TBK1 (Cell Signaling, 51872; 1:1000) antibody, followed by incubation with horseradish peroxidase-conjugated anti-mouse IgG (Jackson Immuno Research Laboratories, 115-035-003; 1:10,000) or anti-rabbit IgG (Invitrogen, G-21234; 1:10,000). The PVDF membranes were then treated with Chemi-Lumi One Super (Nacalai Tesque, 02230–30) to elicit chemiluminescent signals, which were detected and visualized using an LAS-4000 Mini apparatus (GE Healthcare).

### Measurement of virus titers

For measurement of SARS-CoV-2 titer, the bronchoalveolar fluid (BALF) was collected by washing the trachea and lungs of hamsters twice by injecting a total of 2.5 ml low-glucose DMEM (Nacalai Tesque, 08456–65) containing 5% FBS. The virus titer was measured as follows: aliquots of 200 μl of serial 10-fold dilutions of the BALF by low-glucose DMEM containing 5% FBS were inoculated into VeroE6/TMPRSS2 cells in 6-well plates. After 1 hour of incubation, cells were washed with PBS thoroughly and overlaid with 2 ml of agar medium. The number of plaques in each well was counted 2 days after inoculation.

### Knockdown of TRPV4 using shRNA

VeroE6/TMPRSS2 cells were transfected with TRPV4-targeting shRNA (Santa Cruz Biotechnology, sc-61726-SH) using Lipofectamine 2000 (Thermo Fisher Scientific) according to the manufacturer’s protocol. After transfection, cells were selected with puromycin (2 µg/mL) for 2–3 weeks to establish stable TRPV4 knockdown cells. Knockdown efficiency was confirmed by Western blot analysis (Fig 2G).

### LDH release assay

LDH release assays (Promega, G1780) were performed according to the manufacturer’s instructions. LDH release data were used to account for cell death. The data are expressed as a percentage of maximum LDH release.

### Measurement of cytosolic Ca^2+^ levels

VeroE6/TMPRSS2 cells were seeded in black 96-well glass-bottom plates (Matsunami Glass, GP96001) and cultured overnight. The next day, cells were treated with 4 μM of Fluo-8 AM (AAT Bioquest, 21082) in Hanks’ balanced saline solution (HBSS) (Nacalai Tesque, 17460–15) containing 20 mM HEPES (Nacalai Tesque, 17557–94) for 30 min at 37°C. After washing with PBS, the cells were either stimulated for 5 min with the selective TRPV4 agonist GSK1016790A (10 nM), ATP (2 mM), or ionomycin (1 μg/ml), or infected with SARS-CoV-2 variants for 17 hours. Fluorescence intensity was measured using a FLUOstar OPTIMA microplate reader (BMG LABTECH).

### RNA-dependent RNA polymerase (RdRp) activity

SARS-CoV-2 RNA-dependent RNA polymerase (RdRp) activity was measured using a commercially available assay kit (ProFoldin) according to the manufacturer’s instructions with minor modifications. Briefly, reaction mixtures were prepared in 1.5 ml tubes by combining H_2_O, 10 × reaction buffer, 50 × RNA template, 50 × SARS-CoV-2 RdRp enzyme, and 50 × NTPs. The reaction mixtures were incubated at 34°C, 37°C, or 39°C for 2 hours. After incubation, 50 μl of each reaction mixture was transferred to a black 96-well glass-bottom plate (Matsunami Glass, GP96001), followed by the addition of 130 μl of 1 × fluorescence dye per well. Fluorescence intensity was immediately measured using a FLUOstar OPTIMA microplate reader (BMG LABTECH). Background fluorescence from reactions lacking RdRp enzyme was subtracted from all measurements.

### Histopathological examination

Lung tissues were fixed in 10% neutral-buffered formalin, embedded in paraffin using standard procedures, and stained with hematoxylin and eosin (H&E). Pathological scores were evaluated based on histological examination of lung sections collected at 5 days post SARS-CoV-2 infection, as described in S16 Fig.

### Statistical analysis

Statistical significance was tested using nonparametric one-way analysis of variance (ANOVA) with Tukey’s multiple comparison test, non-parametric Mann-Whitney *t* test, or Student’s two-tailed, unpaired t test where indicated in the figure legend, using PRISM software (version 5; GraphPad software). *P* < 0.05 was considered statistically significant.

## Supporting information

S1 Fig Effect of temperature on cytotoxicity in VeroE6/TMPRSS2 cells. VeroE6/TMPRSS2 cells were cultured at 34°C, 37°C, or 39°C overnight. Cytotoxicity was assessed by measuring LDH release in the culture supernatants. Cells treated with lysis buffer served as a positive control. Statistical significance was analyzed by two-way analysis of variance (ANOVA). ***P < 0.001, n.s., not significant.
(PDF)

S2 Fig Replication kinetics of SARS-CoV-2 variants at different temperatures. VeroE6/TMPRSS2 cells were infected with ancestral SARS-CoV-2 (A), Delta variant (B), or Omicron BA.5 variant (C) and cultured at 34°C, 37°C, or 39°C. Viral genomic RNA (gRNA) levels in the supernatants were measured at the indicated time points by quantitative RT-PCR. Each symbol represents an individual value, and bars indicate the mean ± SEM. Statistical significance was analyzed by two-way analysis of variance (ANOVA). *P < 0.05, **P < 0.01, ***P < 0.001, n.s., not significant.
(PDF)

S3 Fig Temperature-dependent replication of SARS-CoV-2 variants is conserved in human respiratory cells. Calu-3 (A-C) or HTEpC cells (D-F) were infected with ancestral SARS-CoV-2 (A and D), Delta variant (B and E), or Omicron BA.5 variant (C and F) and cultured at 34°C, 37°C, or 39°C. Viral genomic RNA (gRNA) levels in the supernatants were measured at the indicated time points by quantitative RT-PCR. Each symbol represents an individual value, and bars indicate the mean ± SEM. Statistical significance was analyzed by two-way analysis of variance (ANOVA). *P < 0.05, **P < 0.01, ***P < 0.001.
(PDF)

S4 Fig RdRp activity is highest at 34°C. SARS-CoV-2 RNA-dependent RNA polymerase (RdRp) activity was measured using a cell-free assay. Reaction mixtures containing RdRp, RNA template, and NTPs were incubated at the indicated temperatures for 2 hours, and RNA synthesis was quantified by fluorescence intensity (RFU). Reactions lacking RdRp enzyme (−) were included as a negative control. Each symbol represents an individual value, and bars indicate the mean ± SEM. Statistical significance was analyzed by two-way analysis of variance (ANOVA). ***P < 0.001.
(PDF)

S5 Fig Effect of a TRPV4 agonist on SARS-CoV-2 replication at different temperatures. (A and B) VeroE6/TMPRSS2 cells were infected with SARS-CoV-2 delta variant in the presence or absence of TRPV4 agonist and cultured at indicated temperatures. Total RNAs were extracted from cell-free supernatants at 24 h p.i. and SARS-CoV-2 N gRNA levels were assessed by quantitative reverse transcription PCR. Each symbol indicates individual values. Statistical significance was analyzed by two-way analysis of variance (ANOVA). n.s., not significant.
(PDF)

S6 Fig Effect of a TRPV4 antagonist on SARS-CoV-2 replication at different temperatures. VeroE6/TMPRSS2 cells were infected with SARS-CoV-2 delta variant in the presence or absence of TRPV4 antagonist and cultured at 34°C (A) or 39°C (B). Total RNAs were extracted from cell-free supernatants at 24 h p.i. and SARS-CoV-2 N gRNA levels were assessed by quantitative reverse transcription PCR. Each symbol indicates individual values. Statistical significance was analyzed by two-way analysis of variance (ANOVA). *P < 0.05, **P < 0.01, ***P < 0.001, n.s., not significant.
(PDF)

S7 Fig TRPV4 is dispensable for replication of a cold-adapted SARS-CoV-2 variant at low temperature. (A) VeroE6/TMPRSS2 cells were infected with parental or cold-adapted SARS-CoV-2 Omicron BA.5 variants and cultured at 21°C. Viral genomic RNA (gRNA) levels in the supernatants were measured by quantitative RT-PCR at the indicated time points. (B and C) Control or TRPV4 knockdown VeroE6/TMPRSS2 cells were infected with the cold-adapted virus and cultured at 21°C. Viral genomic RNA levels were measured at the indicated time points (B). Infectious viral titers in the supernatants from control or TRPV4 knockdown cells infected with the cold-adapted virus were determined by plaque assay (C). Data are presented as mean ± SEM. Statistical significance was analyzed by two-tailed unpaired Student’s t test. n.s., not significant; **P < 0.01, ***P < 0.001.
(PDF)

S8 Fig A selective agonist for TRPV4 receptor induces Ca2+ influx. Fluo-8 AM-treated VeroE6/TMPRSS2 cells were stimulated with the selective TRPV4 agonist GSK1016790A (10 nM) for 5 min. Relative fluorescence units (RFU) were measured at 5 min after stimulation. Statistical significance was analyzed by two-tailed unpaired Student’s t test. ***P < 0.001.
(PDF)

S9 Fig Knockdown of the TRPV4 inhibits GSK1016970A-induced Ca2+ influx. Fluo-8 AM-treated WT or TRPV4-knockdown VeroE6/TMPRSS2 cells were stimulated with 10 nM GSK1016790A (A) or 2 mM ATP (B) for 5 min. Relative fluorescence units (RFU) were measured at 5 min after stimulation. Statistical significance was analyzed by two-tailed unpaired Student’s t test. *P < 0.05.
(PDF)

S10 Fig Infection with SARS-CoV-2 variants induces Ca2+ influx. VeroE6/TMPRSS2 cells were infected with ancestral SARS-CoV-2, Delta variant, or omicron BA.5 variant for 17 hours. Relative fluorescence units (RFU) were measured at 17 hours after infection. Statistical significance was analyzed by two-way analysis of variance (ANOVA). *P < 0.05, **P < 0.01.
(PDF)

S11 Fig Heat-inactivated SARS-CoV-2 fails to induce Ca2+ influx. Fluo-8 AM-treated VeroE6/TMPRSS2 cells were infected with live or heat-inactivated SARS-CoV-2 for 17 hours. Heat inactivation was performed at 100°C for 5 minutes prior to infection. Intracellular Ca^2+^ levels were quantified by measuring relative fluorescence units (RFU). Each symbol represents an individual value. Statistical significance was analyzed by two-way analysis of variance (ANOVA). ***P < 0.001.
(PDF)

S12 Fig TRPV4 knockdown reduces SARS-CoV-2-induced Ca2+ influx. Fluo-8 AM-treated control or TRPV4 knockdown VeroE6/TMPRSS2 cells were infected with SARS-CoV-2 variants for 17 hours. Relative fluorescence units (RFU) were measured at 17 hours after infection. Statistical significance was analyzed by two-way analysis of variance (ANOVA). *P < 0.05, **P < 0.01.
(PDF)

S13 Fig Effect of ionomycin on SARS-CoV-2 replication at different temperatures. VeroE6/TMPRSS2 cells were infected with SARS-CoV-2 delta variant in the presence or absence of ionomycin (1 μg/ml) and cultured at 37°C (A) or 39°C (B). Total RNAs were extracted from cell-free supernatants at 24 h p.i. and SARS-CoV-2 N gRNA levels were assessed by quantitative reverse transcription PCR. Each symbol indicates individual values. Statistical significance was analyzed by two-tailed unpaired Student’s t test. n.s., not significant.
(PDF)

S14 Fig Effect of BAPTA-AM on SARS-CoV-2 replication at different temperatures. VeroE6/TMPRSS2 cells were infected with SARS-CoV-2 delta variant in the presence or absence of BAPTA-AM and cultured at 34°C (A) or 39°C (B). Total RNAs were extracted from cell-free supernatants at 24 h p.i. and SARS-CoV-2 N gRNA levels were assessed by quantitative reverse transcription PCR. Each symbol indicates individual values. Statistical significance was analyzed by two-way analysis of variance (ANOVA). n.s., not significant.
(PDF)

S15 Fig Manidipine inhibits TRPV4-mediated and virus-induced calcium influx. Fluo-8 AM-treated VeroE6/TMPRSS2 cells were stimulated with the selective TRPV4 agonist GSK1016790A (10 nM) or ionomycin (1 μg/ml) for 5 min, or infected with the SARS-CoV-2 Delta or Omicron BA.5 variant for 17 hours. Relative fluorescence units (RFU) were measured after stimulation. Statistical significance was analyzed by two-tailed unpaired Student’s t test. *P < 0.05, **P < 0.01, ***P < 0.001.
(PDF)

S16 Fig Representative histopathological findings and scoring criteria used for evaluation of lung pathology in SARS-CoV-2-infected hamsters. Hamsters were intranasally infected with SARS-CoV-2 and sacrificed at 5 days post-infection. Lung tissues were collected and subjected to hematoxylin and eosin (H&E) staining. A pathological score was assigned based on the sum of the following three histological parameters: (A) Bronchitis/bronchiolitis, characterized by epithelial degeneration and inflammatory cell infiltration in the bronchi and bronchioles; (B) Alveolar infiltration of inflammatory cells, hemorrhage, and pulmonary edema; (C) Subpleural inflammation with activated mesothelial cells. Each category was scored on a scale from 0 to 3, and the total pathological score (maximum 9 points) was used to evaluate the overall severity of lung inflammation. Scale bars: 200 μm.
(PDF)

S1 Raw Gel Uncropped immunoblots corresponding to the immunoblots shown in the main figures. (PDF)

S1 Data Raw data of main figures. (XLSX)

S2 Data Raw data of supporting information figures. (XLSX)
