# Supplementary material for: TRPV4-mediated calcium influx contributes temperature-dependent SARS-CoV-2 replication
Source: PLoS Pathog. 2026 Aug 21;22(8):e1014520. doi: 10.1371/journal.ppat.1014520 (PMC13524343; doi:10.1371/journal.ppat.1014520)
Supplement: Supplementary file 1 [file ppat.1014520.s001.pdf]

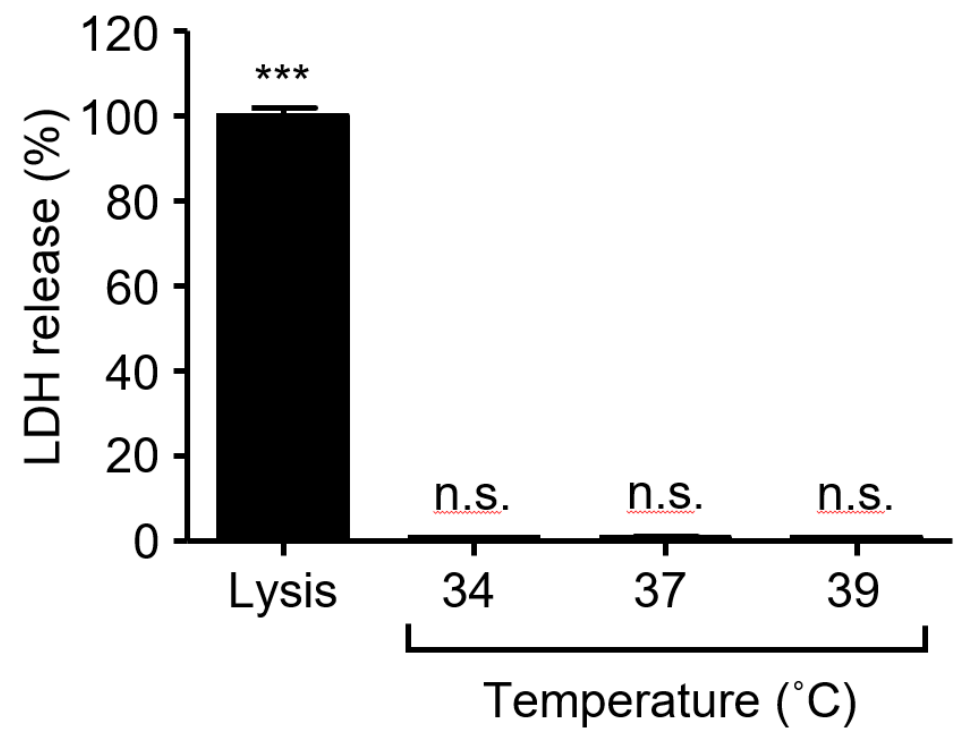

Figure S1. Effect of temperature on cytotoxicity in VeroE6/TMPRSS2 cells. VeroE6/TMPRSS2 cells were cultured at 34°C, 37°C, or 39°C overnight. Cytotoxicity was assessed by measuring LDH release in the culture supernatants. Cells treated with lysis buffer served as a positive control. Statistical significance was analyzed by two-way analysis of variance (ANOVA). ***$P < 0.001$, n.s., not significant.
