# Supplementary material for: TRPV4-mediated calcium influx contributes temperature-dependent SARS-CoV-2 replication
Source: PLoS Pathog. 2026 Aug 21;22(8):e1014520. doi: 10.1371/journal.ppat.1014520 (PMC13524343; doi:10.1371/journal.ppat.1014520)
Supplement: Supplementary file 2 [file ppat.1014520.s002.pdf]

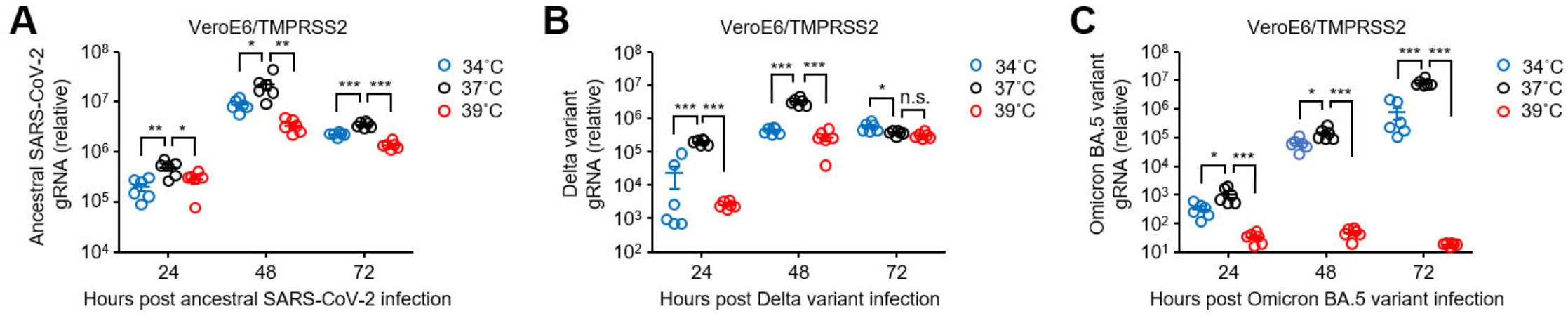

Figure S2. Replication kinetics of SARS-CoV-2 variants at different temperatures.

VeroE6/TMPRSS2 cells were infected with ancestral SARS-CoV-2 (A), Delta variant (B), or Omicron BA.5 variant (C) and cultured at 34°C, 37°C, or 39°C. Viral genomic RNA (gRNA) levels in the supernatants were measured at the indicated time points by quantitative RT-PCR. Each symbol represents an individual value, and bars indicate the mean ± SEM. Statistical significance was analyzed by two-way analysis of variance (ANOVA). **P* < 0.05, ***P* < 0.01, ****P* < 0.001, n.s., not significant.
