# Supplementary material for: TRPV4-mediated calcium influx contributes temperature-dependent SARS-CoV-2 replication
Source: PLoS Pathog. 2026 Aug 21;22(8):e1014520. doi: 10.1371/journal.ppat.1014520 (PMC13524343; doi:10.1371/journal.ppat.1014520)
Supplement: Supplementary file 3 [file ppat.1014520.s003.pdf]

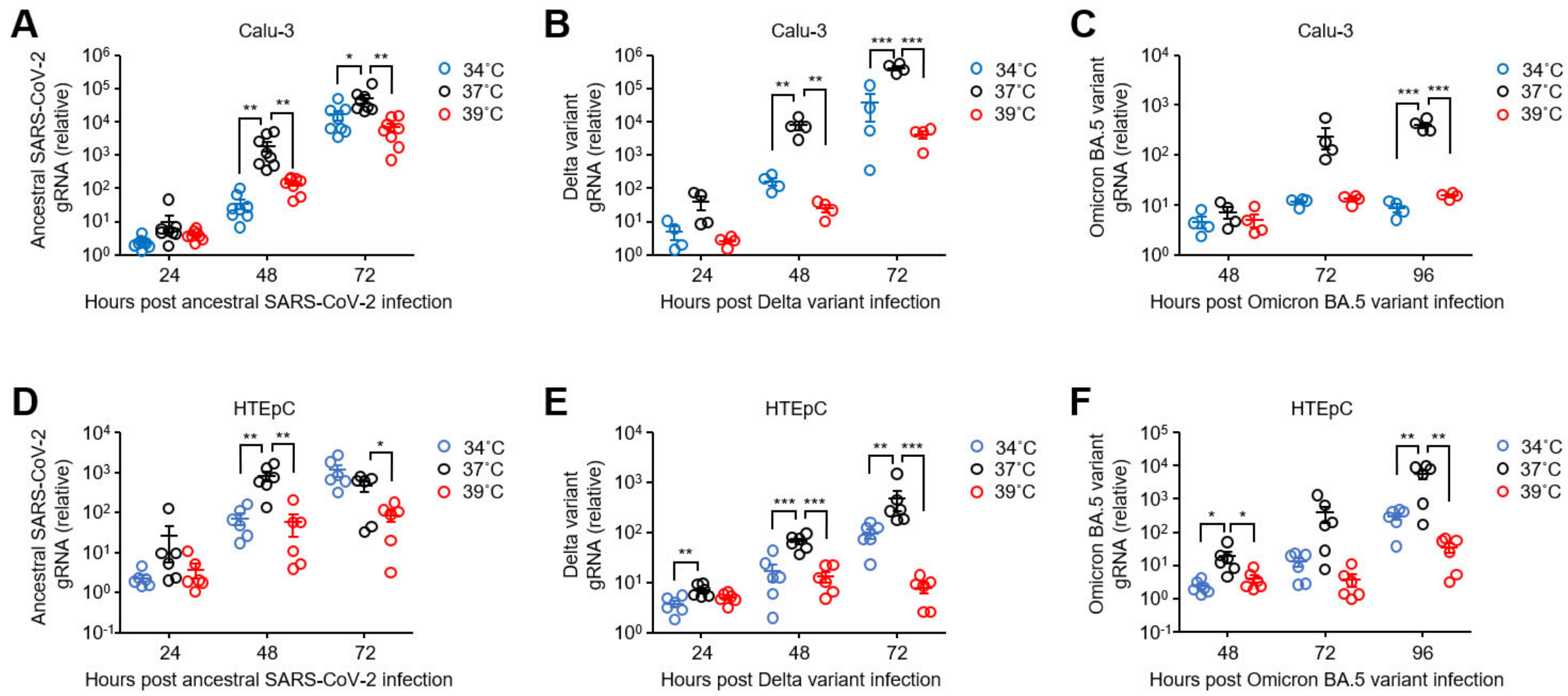

Figure S3. Temperature-dependent replication of SARS-CoV-2 variants is conserved in human respiratory cells.

Calu-3 (A-C) or HTEpC cells (D-F) were infected with ancestral SARS-CoV-2 (A and D), Delta variant (B and E), or Omicron BA.5 variant (C and F) and cultured at 34°C, 37°C, or 39°C. Viral genomic RNA (gRNA) levels in the supernatants were measured at the indicated time points by quantitative RT-PCR. Each symbol represents an individual value, and bars indicate the mean ± SEM. Statistical significance was analyzed by two-way analysis of variance (ANOVA). *$P < 0.05$, **$P < 0.01$, ***$P < 0.001$.
