# Supplementary material for: TRPV4-mediated calcium influx contributes temperature-dependent SARS-CoV-2 replication
Source: PLoS Pathog. 2026 Aug 21;22(8):e1014520. doi: 10.1371/journal.ppat.1014520 (PMC13524343; doi:10.1371/journal.ppat.1014520)
Supplement: Supplementary file 4 [file ppat.1014520.s004.pdf]

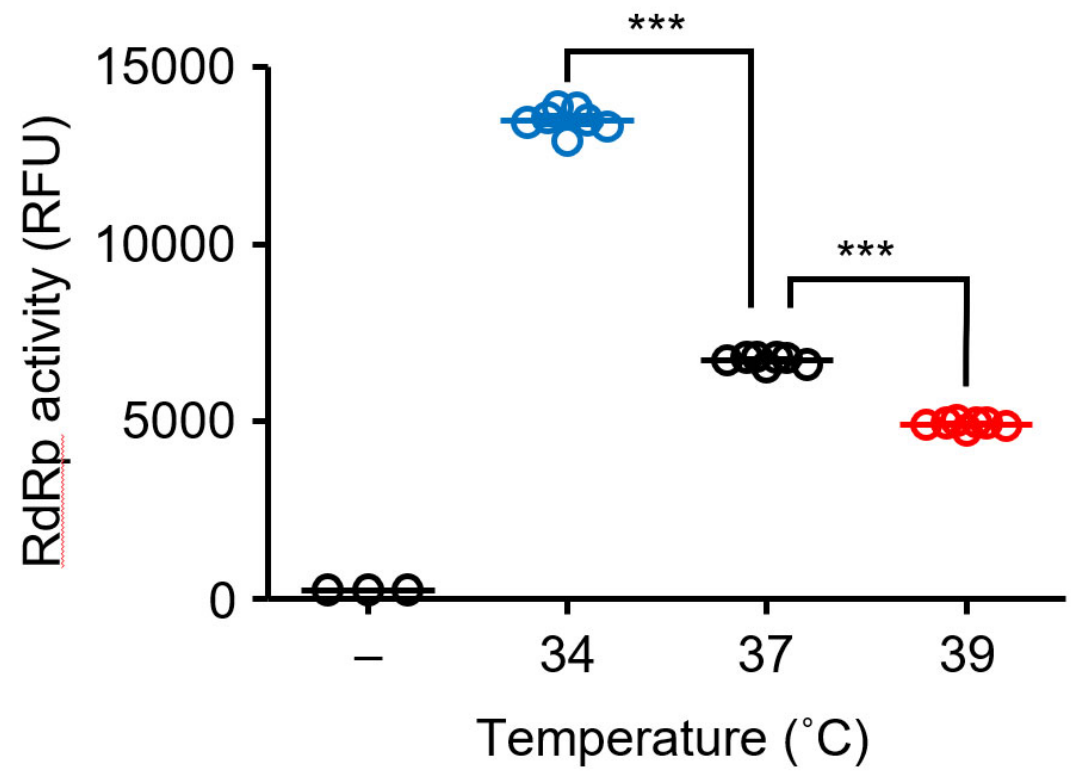

Figure S4. RdRp activity is highest at 34°C.

SARS-CoV-2 RNA-dependent RNA polymerase (RdRp) activity was measured using a cell-free assay. Reaction mixtures containing RdRp, RNA template, and NTPs were incubated at the indicated temperatures for 2 hours, and RNA synthesis was quantified by fluorescence intensity (RFU). Reactions lacking RdRp enzyme (−) were included as a negative control. Each symbol represents an individual value, and bars indicate the mean ± SEM. Statistical significance was analyzed by two-way analysis of variance (ANOVA). ****P* < 0.001.
