# Supplementary material for: TRPV4-mediated calcium influx contributes temperature-dependent SARS-CoV-2 replication
Source: PLoS Pathog. 2026 Aug 21;22(8):e1014520. doi: 10.1371/journal.ppat.1014520 (PMC13524343; doi:10.1371/journal.ppat.1014520)
Supplement: Supplementary file 5 [file ppat.1014520.s005.pdf]

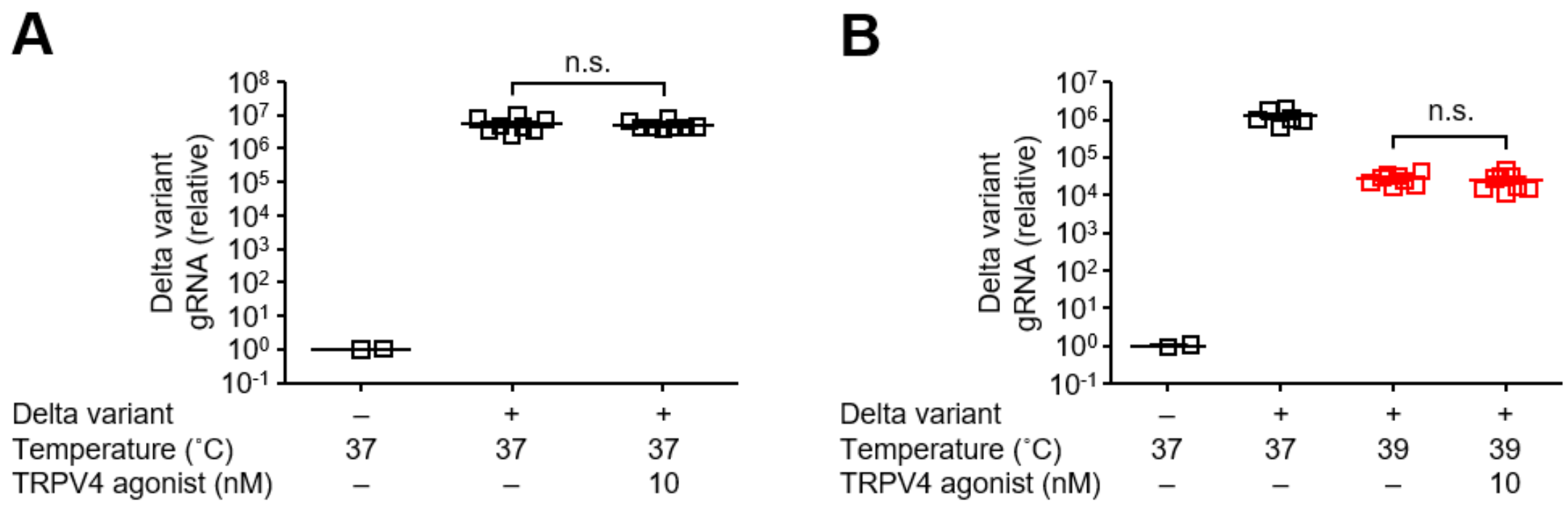

Figure S5. Effect of a TRPV4 agonist on SARS-CoV-2 replication at different temperatures.

(A and B) VeroE6/TMPRSS2 cells were infected with SARS-CoV-2 delta variant in the presence or absence of TRPV4 agonist and cultured at indicated temperatures. Total RNAs were extracted from cell-free supernatants at 24 h p.i. and SARS-CoV-2 N gRNA levels were assessed by quantitative reverse transcription PCR. Each symbol indicates individual values. Statistical significance was analyzed by two-way analysis of variance (ANOVA). n.s., not significant.
