# Supplementary material for: TRPV4-mediated calcium influx contributes temperature-dependent SARS-CoV-2 replication
Source: PLoS Pathog. 2026 Aug 21;22(8):e1014520. doi: 10.1371/journal.ppat.1014520 (PMC13524343; doi:10.1371/journal.ppat.1014520)
Supplement: Supplementary file 7 [file ppat.1014520.s007.pdf]

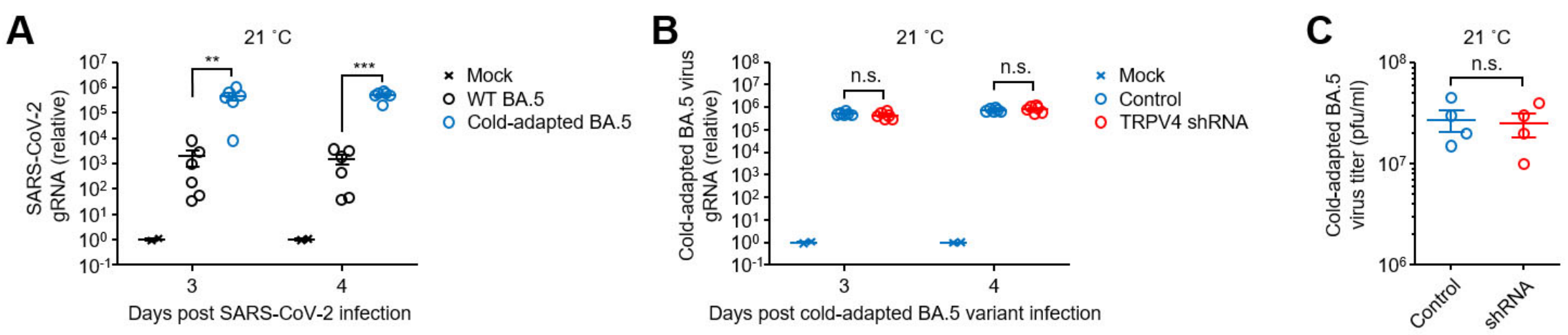


**Figure S7. TRPV4 is dispensable for replication of a cold-adapted SARS-CoV-2 variant at low temperature.**

(A) VeroE6/TMPRSS2 cells were infected with parental or cold-adapted SARS-CoV-2 Omicron BA.5 variants and cultured at 21°C. Viral genomic RNA (gRNA) levels in the supernatants were measured by quantitative RT-PCR at the indicated time points. (B and C) Control or TRPV4 knockdown VeroE6/TMPRSS2 cells were infected with the cold-adapted virus and cultured at 21°C. Viral genomic RNA levels were measured at the indicated time points (B). Infectious viral titers in the supernatants from control or TRPV4 knockdown cells infected with the cold-adapted virus were determined by plaque assay (C). Data are presented as mean ± SEM. Statistical significance was analyzed by two-tailed unpaired Student's t test. n.s., not significant; $^{**}P < 0.01$, $^{***}P < 0.001$.
