# Supplementary material for: TRPV4-mediated calcium influx contributes temperature-dependent SARS-CoV-2 replication
Source: PLoS Pathog. 2026 Aug 21;22(8):e1014520. doi: 10.1371/journal.ppat.1014520 (PMC13524343; doi:10.1371/journal.ppat.1014520)
Supplement: Supplementary file 8 [file ppat.1014520.s008.pdf]

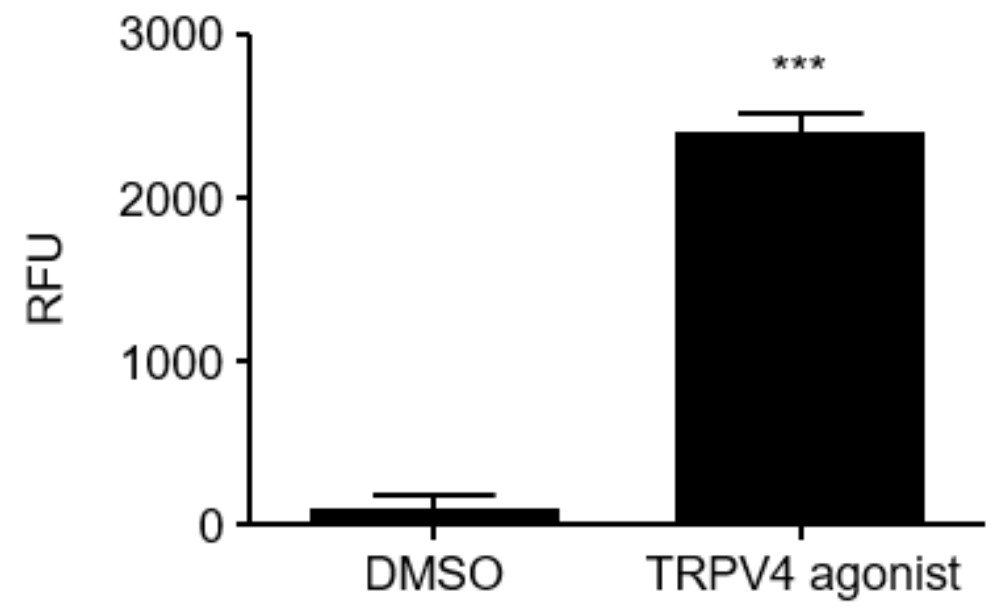

Figure S8. A selective agonist for TRPV4 receptor induces Ca^{2+} influx. Fluo-8 AM-treated VeroE6/TMPRSS2 cells were stimulated with the selective TRPV4 agonist GSK1016790A (10 nM) for 5 min. Relative fluorescence units (RFU) were measured at 5 min after stimulation. Statistical significance was analyzed by two-tailed unpaired Student's t test. ***$P < 0.001$.
