# Supplementary material for: TRPV4-mediated calcium influx contributes temperature-dependent SARS-CoV-2 replication
Source: PLoS Pathog. 2026 Aug 21;22(8):e1014520. doi: 10.1371/journal.ppat.1014520 (PMC13524343; doi:10.1371/journal.ppat.1014520)
Supplement: Supplementary file 9 [file ppat.1014520.s009.pdf]

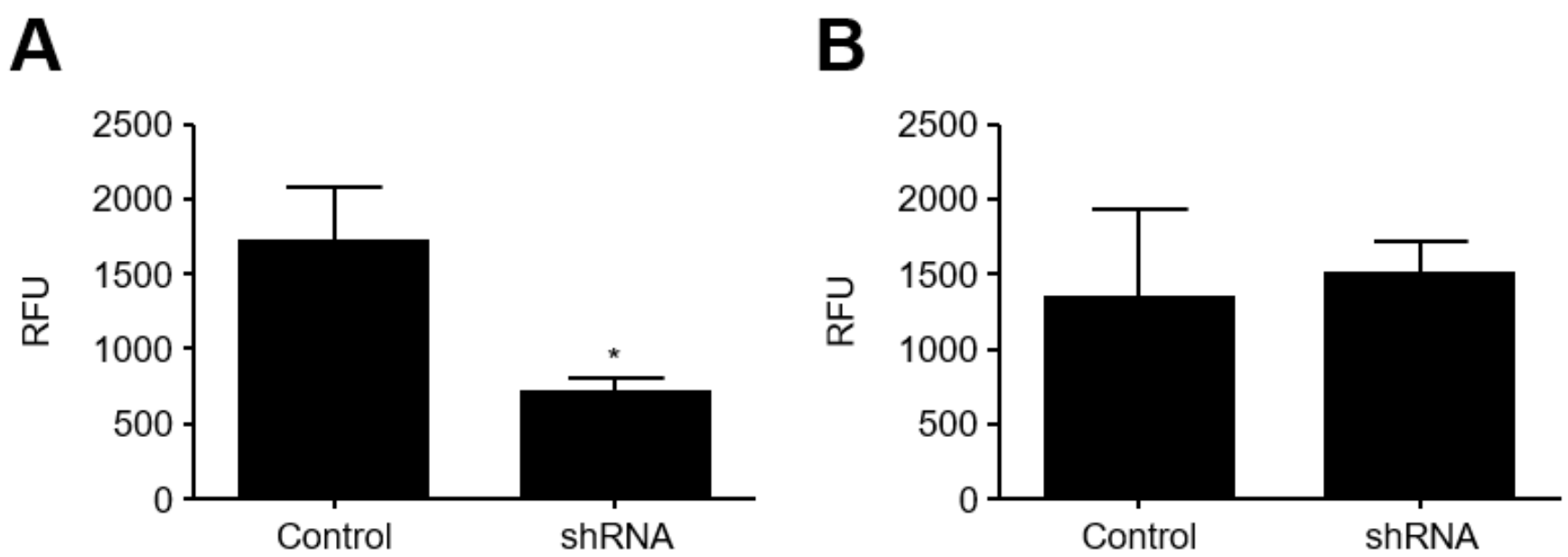

Figure S9. Knockdown of the TRPV4 inhibits GSK1016970A-induced Ca^{2+} influx.

Fluo-8 AM-treated WT or TRPV4-knockdown VeroE6/TMPRSS2 cells were stimulated with 10 nM GSK1016790A (A) or 2 mM ATP (B) for 5 min. Relative fluorescence units (RFU) were measured at 5 min after stimulation. Statistical significance was analyzed by two-tailed unpaired Student's t test. *$P < 0.05$.
