# Supplementary material for: TRPV4-mediated calcium influx contributes temperature-dependent SARS-CoV-2 replication
Source: PLoS Pathog. 2026 Aug 21;22(8):e1014520. doi: 10.1371/journal.ppat.1014520 (PMC13524343; doi:10.1371/journal.ppat.1014520)
Supplement: Supplementary file 10 [file ppat.1014520.s010.pdf]

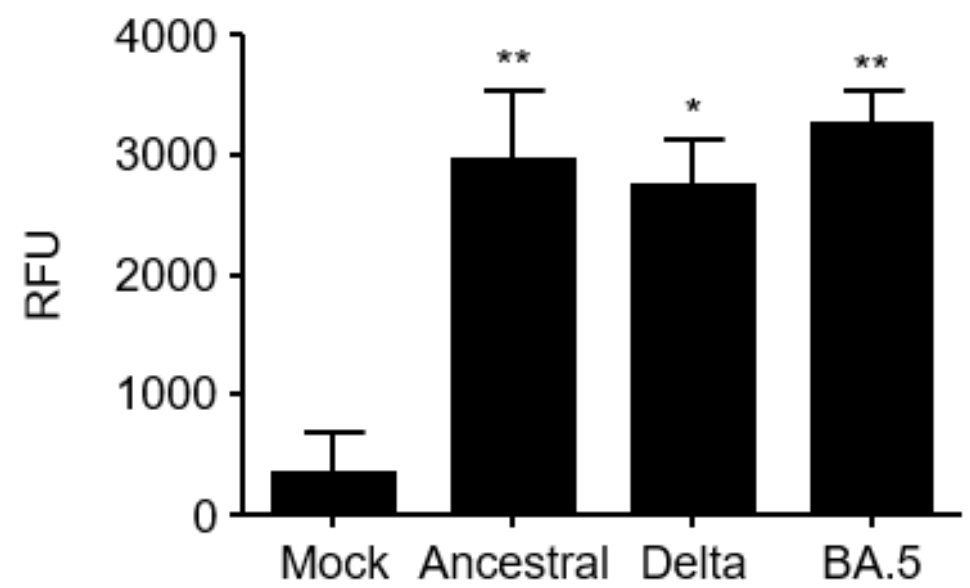

Figure S10. Infection with SARS-CoV-2 variants induces Ca^{2+} influx.
VeroE6/TMPRSS2 cells were infected with ancestral SARS-CoV-2, Delta variant, or omicron BA.5 variant for 17 hours. Relative fluorescence units (RFU) were measured at 17 hours after infection. Statistical significance was analyzed by two-way analysis of variance (ANOVA). *$P < 0.05$, **$P < 0.01$.
