# Supplementary material for: TRPV4-mediated calcium influx contributes temperature-dependent SARS-CoV-2 replication
Source: PLoS Pathog. 2026 Aug 21;22(8):e1014520. doi: 10.1371/journal.ppat.1014520 (PMC13524343; doi:10.1371/journal.ppat.1014520)
Supplement: Supplementary file 11 [file ppat.1014520.s011.pdf]

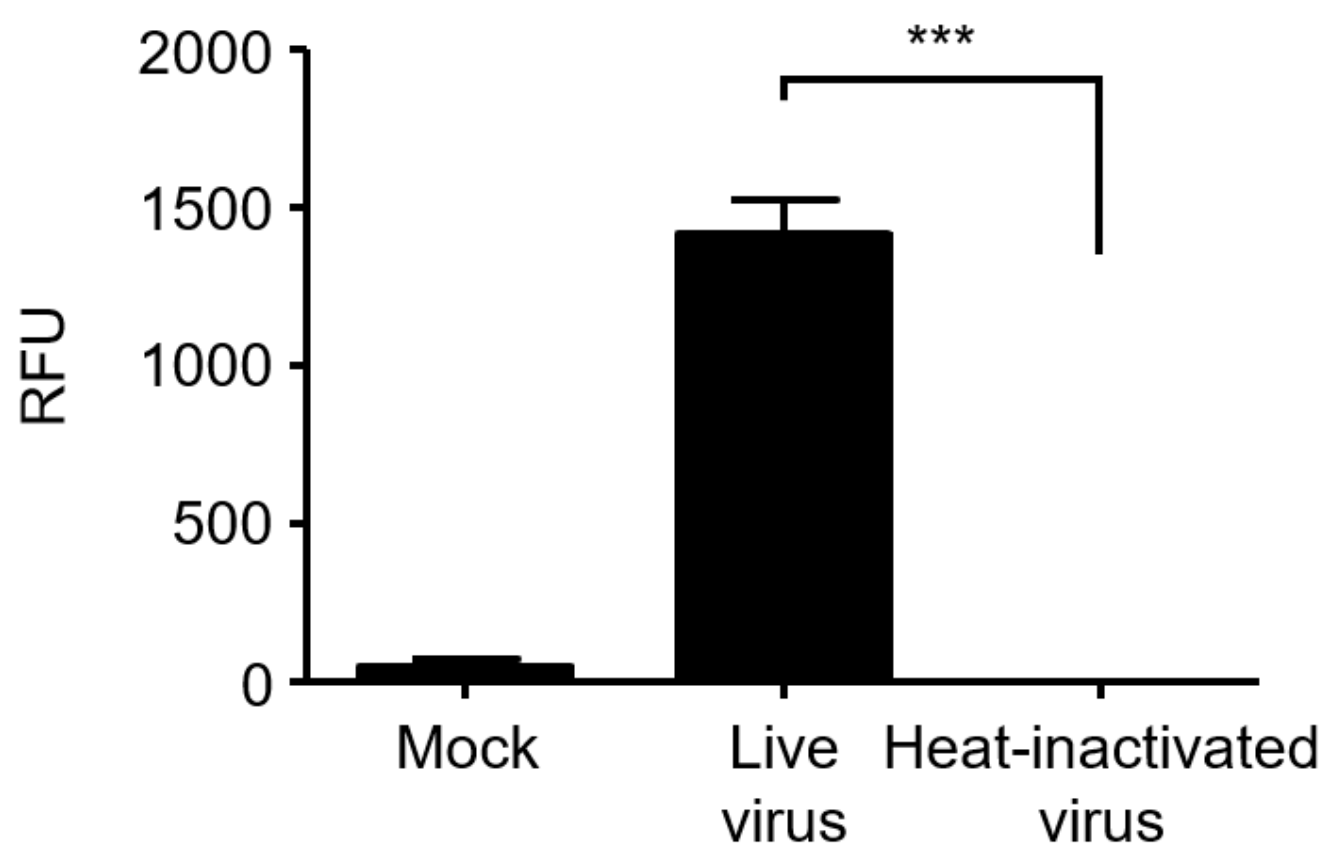

Figure S11. Heat-inactivated SARS-CoV-2 fails to induce Ca^{2+} influx.
Fluo-8 AM-treated VeroE6/TMPRSS2 cells were infected with live or heat-inactivated SARS-CoV-2 for 17 hours. Heat inactivation was performed at 100°C for 5 minutes prior to infection. Intracellular Ca^{2+} levels were quantified by measuring relative fluorescence units (RFU). Each symbol represents an individual value. Statistical significance was analyzed by two-way analysis of variance (ANOVA). ***$P < 0.001$.
