# Supplementary material for: TRPV4-mediated calcium influx contributes temperature-dependent SARS-CoV-2 replication
Source: PLoS Pathog. 2026 Aug 21;22(8):e1014520. doi: 10.1371/journal.ppat.1014520 (PMC13524343; doi:10.1371/journal.ppat.1014520)
Supplement: Supplementary file 12 [file ppat.1014520.s012.pdf]

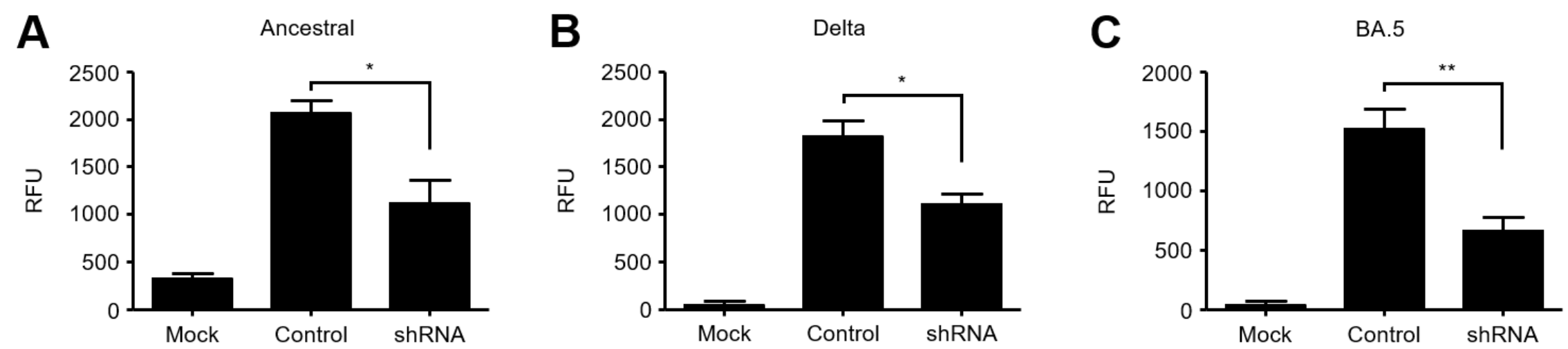

Figure S12. TRPV4 knockdown reduces SARS-CoV-2-induced Ca^{2+} influx. Fluo-8 AM-treated control or TRPV4 knockdown VeroE6/TMPRSS2 cells were infected with SARS-CoV-2 variants for 17 hours. Relative fluorescence units (RFU) were measured at 17 hours after infection. Statistical significance was analyzed by two-way analysis of variance (ANOVA). *$P < 0.05$, **$P < 0.01$.
