# Supplementary material for: TRPV4-mediated calcium influx contributes temperature-dependent SARS-CoV-2 replication
Source: PLoS Pathog. 2026 Aug 21;22(8):e1014520. doi: 10.1371/journal.ppat.1014520 (PMC13524343; doi:10.1371/journal.ppat.1014520)
Supplement: Supplementary file 13 [file ppat.1014520.s013.pdf]

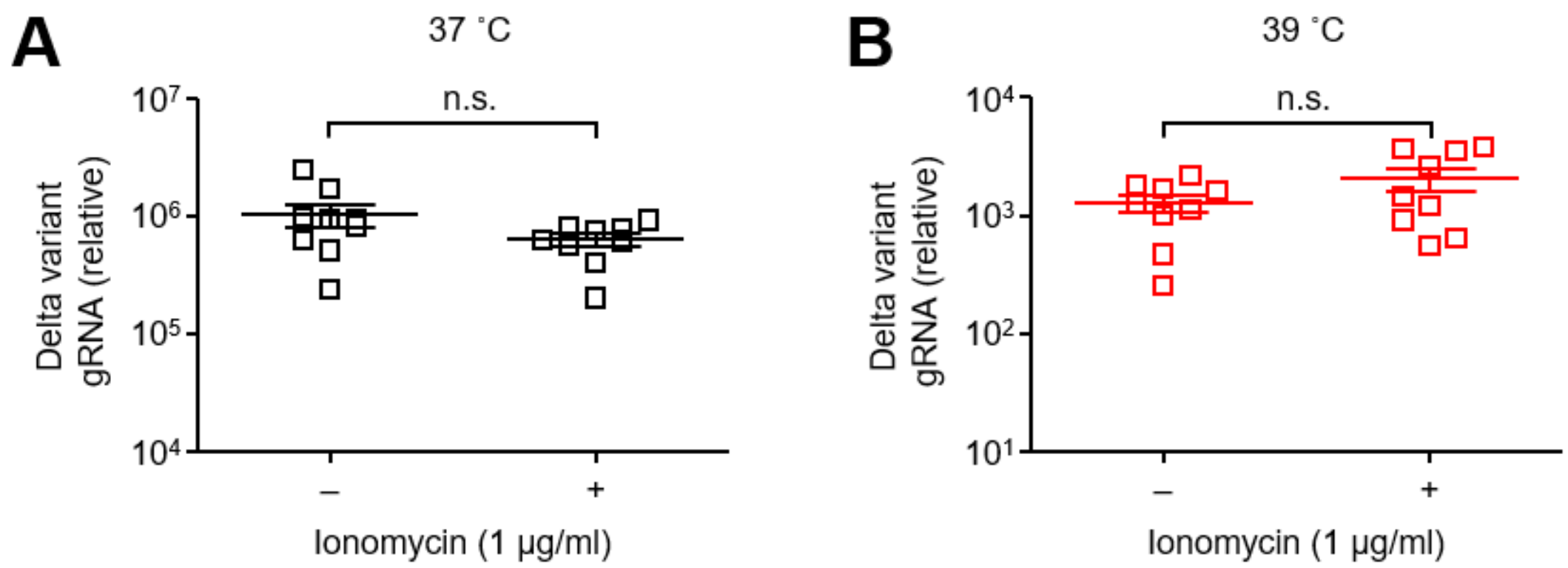

Figure S13. Effect of ionomycin on SARS-CoV-2 replication at different temperatures.

VeroE6/TMPRSS2 cells were infected with SARS-CoV-2 delta variant in the presence or absence of ionomycin (1 µg/ml) and cultured at 37°C (A) or 39°C (B). Total RNAs were extracted from cell-free supernatants at 24 h p.i. and SARS-CoV-2 N gRNA levels were assessed by quantitative reverse transcription PCR. Each symbol indicates individual values. Statistical significance was analyzed by two-tailed unpaired Student’s t test. n.s., not significant.
