# Supplementary material for: TRPV4-mediated calcium influx contributes temperature-dependent SARS-CoV-2 replication
Source: PLoS Pathog. 2026 Aug 21;22(8):e1014520. doi: 10.1371/journal.ppat.1014520 (PMC13524343; doi:10.1371/journal.ppat.1014520)
Supplement: Supplementary file 15 [file ppat.1014520.s015.pdf]

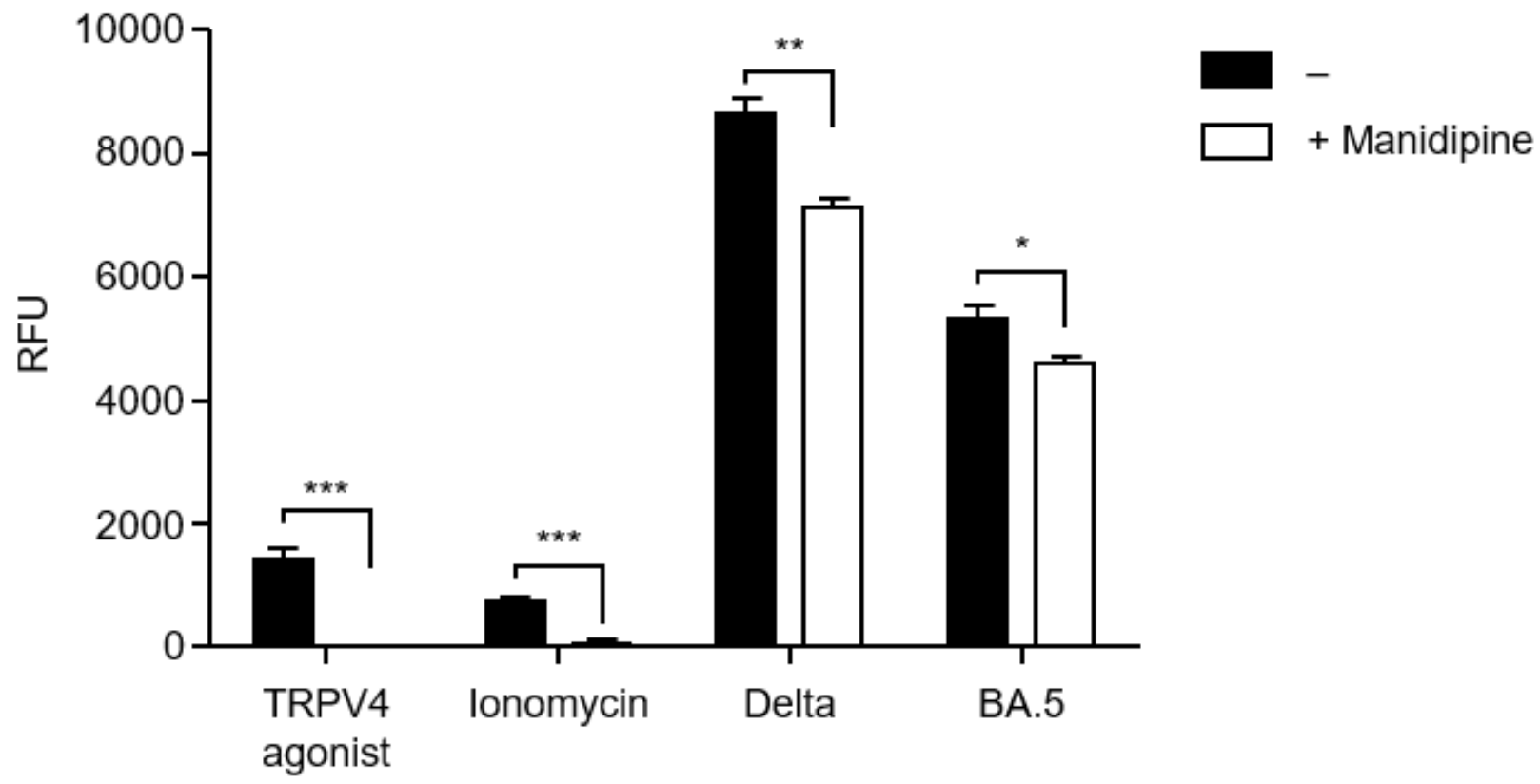

Figure S15. Manidipine inhibits TRPV4-mediated and virus-induced calcium influx.

Fluo-8 AM-treated VeroE6/TMPRSS2 cells were stimulated with the selective TRPV4 agonist GSK1016790A (10 nM) or ionomycin (1 µg/ml) for 5 min, or infected with the SARS-CoV-2 Delta or Omicron BA.5 variant for 17 hours. Relative fluorescence units (RFU) were measured after stimulation. Statistical significance was analyzed by two-tailed unpaired Student’s t test. *$P < 0.05$, **$P < 0.01$, ***$P < 0.001$.
