# Supplementary material for: TRPV4-mediated calcium influx contributes temperature-dependent SARS-CoV-2 replication
Source: PLoS Pathog. 2026 Aug 21;22(8):e1014520. doi: 10.1371/journal.ppat.1014520 (PMC13524343; doi:10.1371/journal.ppat.1014520)
Supplement: Supplementary file 16 [file ppat.1014520.s016.pdf]

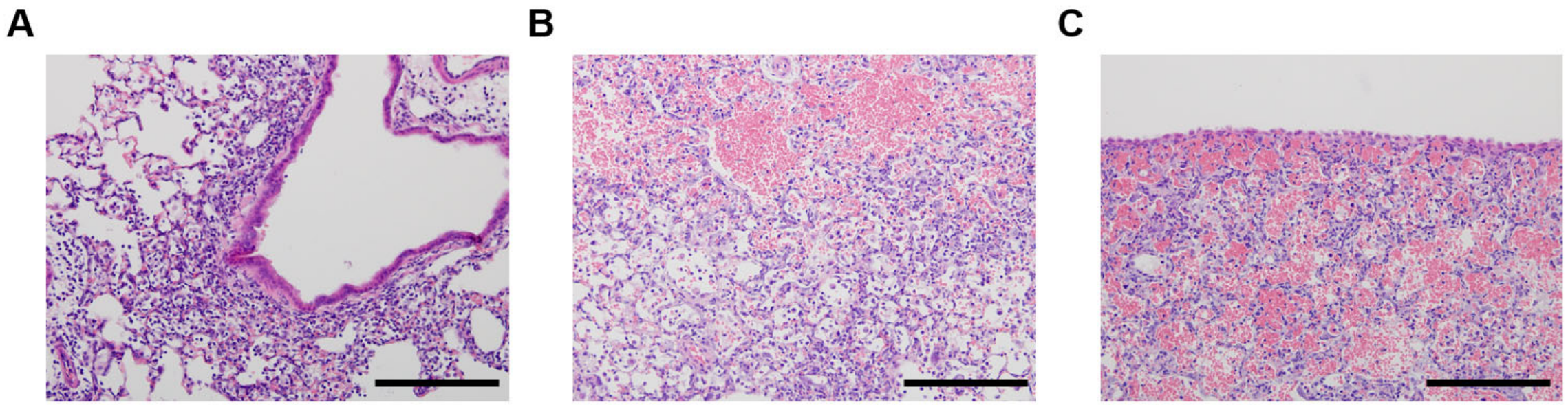

Figure S16. Representative histopathological findings and scoring criteria used for evaluation of lung pathology in SARS-CoV-2-infected hamsters. Hamsters were intranasally infected with SARS-CoV-2 and sacrificed at 5 days post-infection. Lung tissues were collected and subjected to hematoxylin and eosin (H&E) staining. A pathological score was assigned based on the sum of the following three histological parameters: (A) Bronchitis/bronchiolitis, characterized by epithelial degeneration and inflammatory cell infiltration in the bronchi and bronchioles; (B) Alveolar infiltration of inflammatory cells, hemorrhage, and pulmonary edema; (C) Subpleural inflammation with activated mesothelial cells. Each category was scored on a scale from 0 to 3, and the total pathological score (maximum 9 points) was used to evaluate the overall severity of lung inflammation. Scale bars: 200 μm.
