# Supplementary material for: TRPV4-mediated calcium influx contributes temperature-dependent SARS-CoV-2 replication
Source: PLoS Pathog. 2026 Aug 21;22(8):e1014520. doi: 10.1371/journal.ppat.1014520 (PMC13524343; doi:10.1371/journal.ppat.1014520)
Supplement: Supplementary file 17 [file ppat.1014520.s017.pdf]

Fig .2G

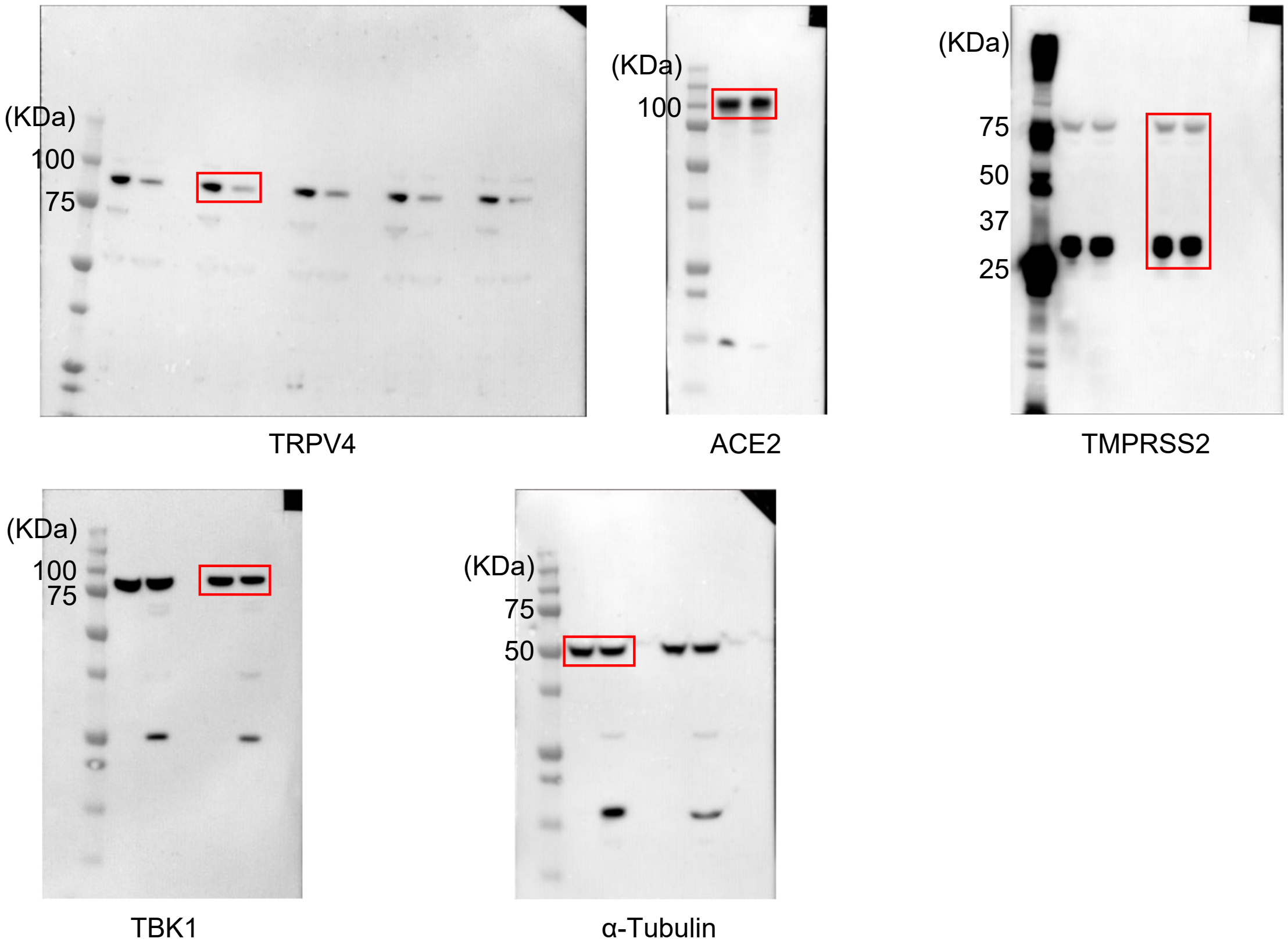

Fig .4C

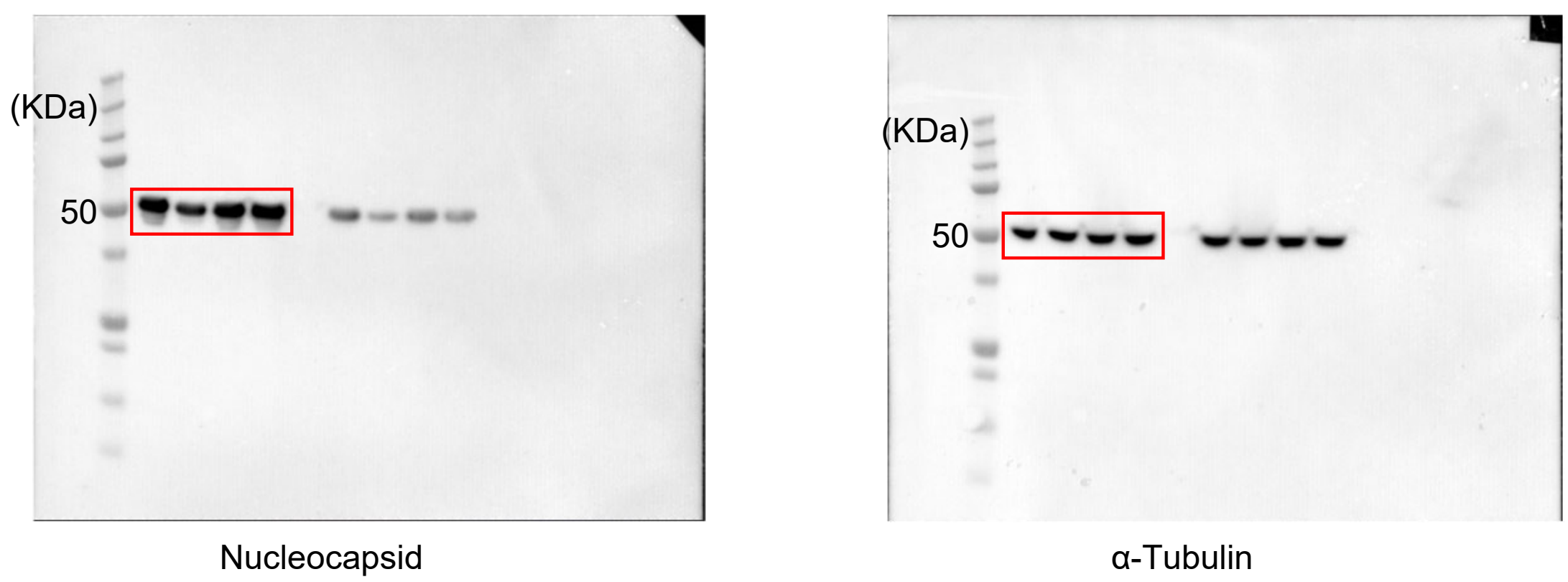

Fig .4G

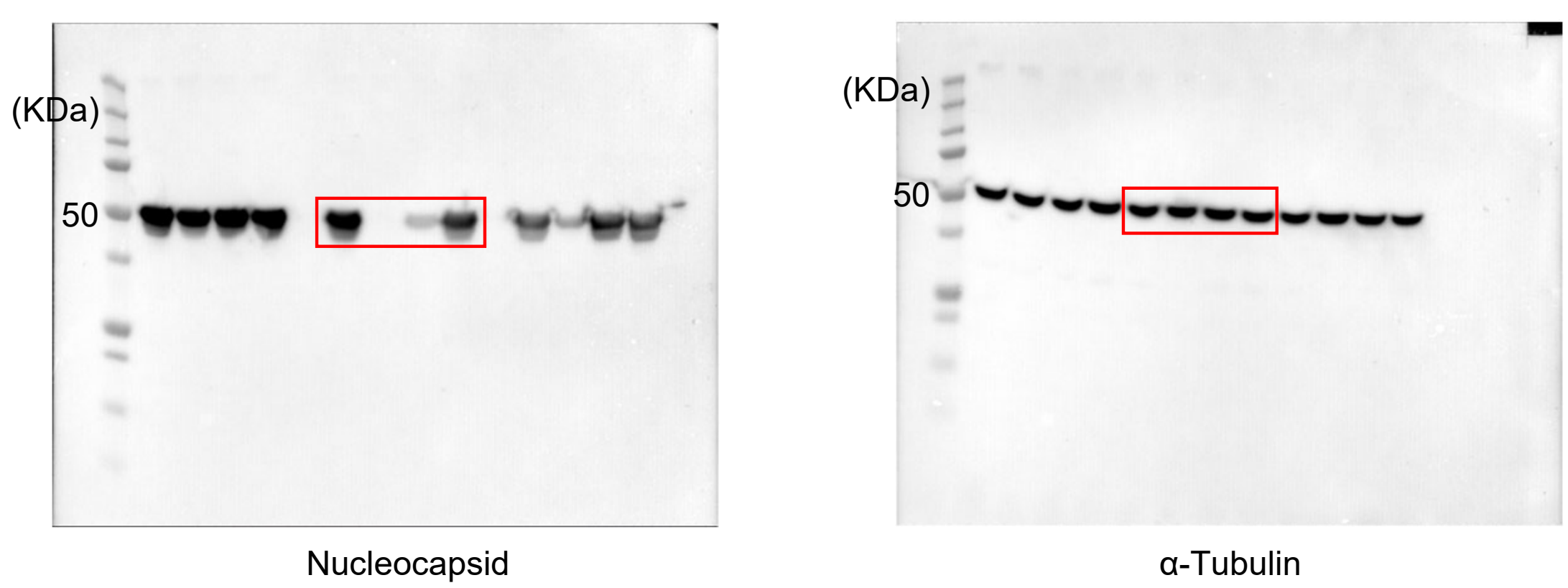

Fig .5C

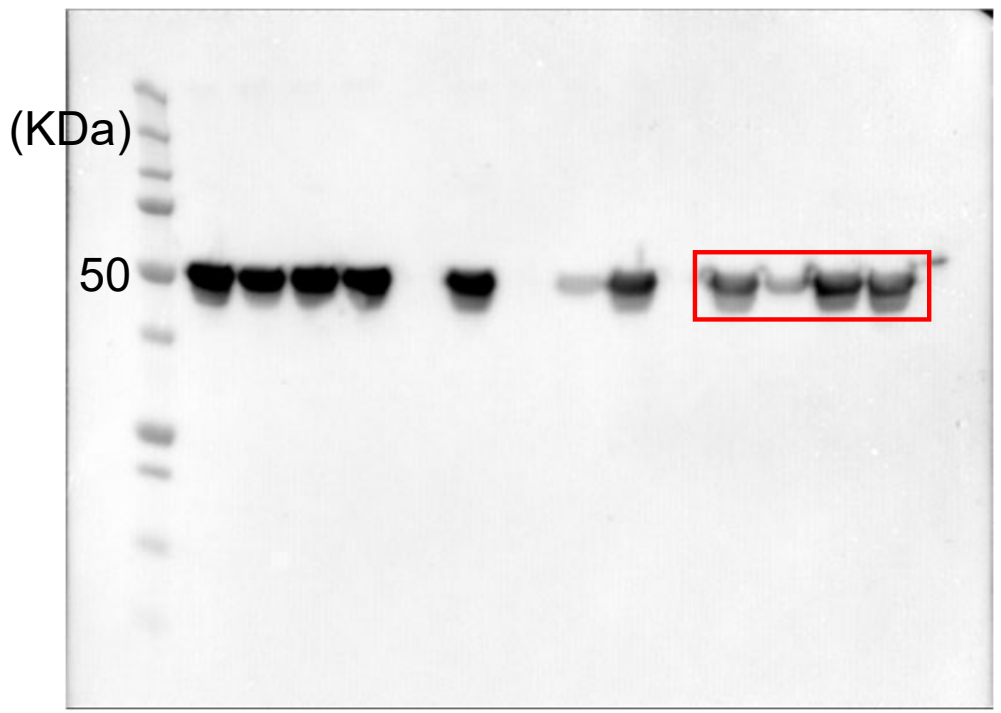

Nucleocapsid

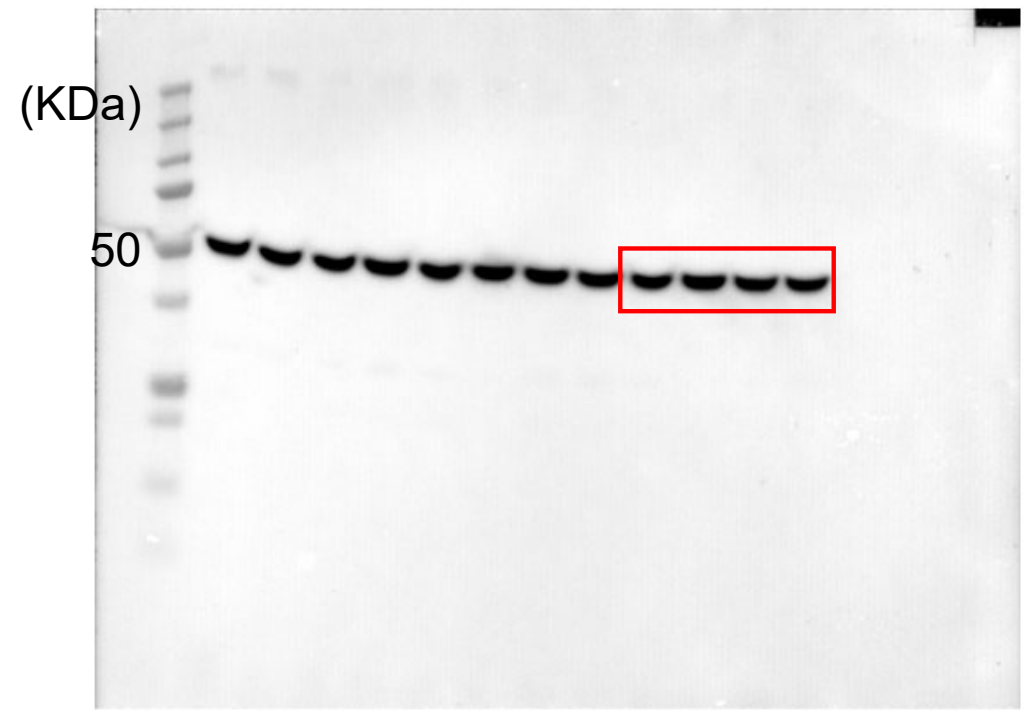

α-Tubulin
