# Supplementary material for: TRPV4-mediated calcium influx contributes temperature-dependent SARS-CoV-2 replication
Source: PLoS Pathog. 2026 Aug 21;22(8):e1014520. doi: 10.1371/journal.ppat.1014520 (PMC13524343; doi:10.1371/journal.ppat.1014520)
Supplement: Supplementary file 19 [file ppat.1014520.s021.pdf]

Additional Editor Comments (if provided):

Thank you for submitting the revised version of your manuscript. The revised manuscript has now been evaluated by the reviewers.

The reviewers acknowledge that the revision has addressed many of the concerns raised previously. In particular, the evidence supporting a role for intracellular calcium in SARS-CoV-2 replication and TRPV4 as a contributor to this process is considered convincing. However, concerns remain regarding the central mechanistic conclusion that the TRPV4–Ca^{2+} axis underlies temperature-dependent SARS-CoV-2 replication.

In preparing your revision, please carefully consider whether the overall framing and conclusions of the manuscript are fully supported by the experimental evidence. If the current data do not directly establish the mechanistic link between temperature and the TRPV4–Ca^{2+} axis, the title, abstract, Results, and Discussion should be revised accordingly to ensure that the conclusions accurately reflect the scope of the data. Please also address all reviewer comments in a detailed point-by-point response.

We sincerely thank the Editor for this important and thoughtful comment. As suggested, we carefully reconsidered the overall framing of the manuscript to ensure that our conclusions accurately reflect the experimental evidence. We agree that our data support an important contribution of TRPV4-mediated calcium influx to temperature-dependent SARS-CoV-2 replication, but do not definitively establish that the TRPV4–Ca^{2+} axis is the sole or complete mechanism underlying this phenomenon.

Accordingly, we have revised the title, Abstract, Results, and Discussion throughout the manuscript to avoid overstating the mechanistic conclusions. Specifically, we replaced wording implying that TRPV4-mediated calcium influx underlies temperature-dependent SARS-CoV-2 replication with more appropriately qualified language such as "contributes to" or "an important contributor." We also explicitly state in the Discussion and Conclusion that additional temperature-sensitive host factors are likely to participate and that further studies will be required to define the complete molecular mechanism underlying temperature-dependent viral replication.

These revisions are highlighted in yellow in the revised manuscript. We believe that the revised manuscript now more accurately reflects the scope of the experimental data while preserving the major findings of the study.

Reviewers' Comments:

Reviewer's Responses to Questions

Part I - Summary

Reviewer #1: I sincerely thank the authors for thoroughly addressing all of my concerns.

We sincerely thank the reviewer for the positive and encouraging comments. We greatly appreciate your careful evaluation of our revised manuscript and are delighted that our revisions have satisfactorily addressed all of your concerns.

Reviewer #2: Kobayashi and Abe et al. identify TRPV4, a temperature-dependent calcium channel, as a major contributor to the temperature-dependent replication of SARS-CoV-2 at physiologically relevant temperatures: 34°C, representing the upper respiratory tract; 38°C, representing core body temperature; and 39°C, representing a fever-like condition. The authors show consistent trends across three viral variants. Their mechanistic evidence is supported by a comprehensive set of experiments focused on calcium influx. Specifically, they use a TRPV4 agonist at 34°C to increase viral replication and a TRPV4 antagonist at 37°C to inhibit replication. To further support calcium influx as a key factor in temperature-sensitive SARS-CoV-2 replication, they use the broad calcium ionophore ionomycin and the calcium chelator BAPTA-AM to manipulate intracellular calcium levels, producing results consistent with the TRPV4 experiments. The controls are thorough and well designed, including assessments of drug toxicity, potential off-target effects on proteins known to affect SARS-CoV-2 infection such as ACE2 and TMPRSS2, and treatment effects at the relevant temperatures. In addition, the authors perform genetic siRNA knockdown experiments and show that SARS-CoV-2 replication is reduced following TRPV4 knockdown at 37°C. The authors also perform in vivo experiments in a hamster model. Treatment with the general calcium channel blockers cyclosporin A and manidipine has a significant effect on survival. In contrast, a TRPV4-specific inhibitor shows only a modest protective effect in animals. Overall, the data are well controlled and carefully executed, with substantial experimental replication.

We sincerely thank the reviewer for the careful evaluation of our work and for the positive assessment of our revised manuscript. We greatly appreciate your recognition of the extensive experimental validation, including the pharmacological, genetic, and in vivo studies, as well as your acknowledgment of the thorough controls and experimental

design. Your encouraging comments are greatly appreciated and have been invaluable in improving the quality and clarity of our manuscript. In response to the Editor's and reviewer's comments, we have further refined the framing of the manuscript to ensure that our conclusions accurately reflect the scope of the experimental evidence.

Reviewer #3: This manuscript identify the TRPV4 play an important role during the SARS-CoV-2 infection cycle by regulation calcium concentration as the thermosensitive channel. Meanwhile, the authors tested the therapeutic potential of targeting calcium channel in hamster. However, the mechanistic interaction between the TRPV4 and SARS-CoV-2 need further clarification and validation. There have been one review about the host cell calcium dynamics and SARS-CoV-2 infection (https://doi.org/10.1016/j.ceca.2022.102637). Several issues should be further explored or clarification.

We sincerely thank the reviewer for the careful evaluation of our manuscript and for the constructive comments. We appreciate the suggestion to consider the recent review on host cell calcium dynamics during SARS-CoV-2 infection. We have now cited this review in the revised manuscript (Ref#26) and expanded the Discussion to better place our findings in the context of current knowledge regarding calcium signaling during SARS-CoV-2 infection (lines 299-302 page 15). We have also carefully addressed each of the specific comments below. In addition, consistent with the Editor's recommendation, we have further revised the manuscript to moderate the mechanistic interpretation and ensure that our conclusions accurately reflect the experimental evidence.

Part II – Major Issues: Key Experiments Required for Acceptance

Reviewer #1: No additional experiments required from my side.

We sincerely thank the reviewer for this positive assessment and are pleased that no additional experiments are required.

Reviewer #2: n/a

We thank the reviewer for indicating that no additional major experiments are required.

Reviewer #3: The authors should be cautious with the quantification results of viral N gRNA, usually the RNA copies are hundreds or thousands times higher than the viral titers, but it is opposite in this study.

We thank the reviewer for this helpful comment. The viral genomic RNA (gRNA) data

presented in this study represent relative RT-qPCR values normalized to mock-infected controls, rather than absolute RNA copy numbers. Therefore, these values are not directly comparable to infectious virus titers determined by plaque assay. To avoid potential misunderstanding, we have clarified this point in the Materials and Methods section (lines 467-471 page 22).

The authors investigated SARS-CoV-2 replication under different temperature (34 °C ,37°C , and 39 °C) ; however, the impact of temperature on cellular state was neither measured nor discussed, as most mammalian cell lines exhibit growth limitations at 39°C. Notably, it is worth noting that both TRPV4 agonists and inhibitors had minimal effects on SARS-CoV-2 replication.

We thank the reviewer for this helpful suggestion. To address this concern, we measured LDH release from VeroE6/TMPRSS2 cells cultured at 34°C, 37°C, and 39°C. No significant differences in LDH release were observed among the three temperature conditions (S1 Fig), indicating that the reduced viral replication observed at 39°C is unlikely to be attributable to overt temperature-induced cytotoxicity. These new data have been included in the revised manuscript (lines 96-98 page 6, lines 338-341 page 16). In addition, we have added a statement in the Discussion acknowledging that temperature may affect additional cellular processes beyond TRPV4 signaling (lines 381-384 page 18). We believe that these revisions more appropriately reflect the scope of our experimental findings.

Regarding the relatively modest effects of the TRPV4 agonist and antagonist under certain conditions, we believe these findings are consistent with our revised interpretation that TRPV4 is an important contributor, but not the sole determinant, of temperature-dependent SARS-CoV-2 replication. This interpretation has been further emphasized throughout the revised manuscript.

It is interesting to sequence the genome of the cold-adapted SARS-CoV-2, then check the sequence mutations of the cold-adapted variants and confirm the locations of the mutations. This may help us identify which proteins play important functions during the temperature-dependent process. The authors should discuss this point in the manuscript.

We thank the reviewer for this valuable suggestion. We agree that identifying the mutations acquired during cold adaptation would provide important insights into the viral determinants of temperature-dependent replication. However, comprehensive genome sequencing and functional characterization of individual mutations are beyond the scope of the present study, which focuses primarily on the role of host TRPV4-mediated calcium

signaling in temperature-dependent SARS-CoV-2 replication. As suggested, we have now expanded the Discussion to acknowledge this important future direction and to state that identifying the mutations responsible for cold adaptation will be an important subject for future investigation (lines 329-332 page 16).

In the Figure 2E and 3F, the virus titer value of PFU for the control group looks same, are they independent experiments or use the same control?

We thank the reviewer for this careful observation. We confirmed that the experiments shown in Fig. 2E and Fig. 3F were performed as completely independent experiments, each with its own independently generated control group. The apparent similarity in the control virus titers is coincidental and reflects the good reproducibility of the plaque assay rather than the reuse of the same control samples. No data were reused between these experiments.
