# Supplementary material for: TRPV4-mediated calcium influx contributes temperature-dependent SARS-CoV-2 replication
Source: PLoS Pathog. 2026 Aug 21;22(8):e1014520. doi: 10.1371/journal.ppat.1014520 (PMC13524343; doi:10.1371/journal.ppat.1014520)
Supplement: Supplementary file 20 [file ppat.1014520.s020.pdf]

Reviewer 1 comments:

This manuscript addresses an important question regarding the temperature dependence of SARS-CoV-2 replication and the potential involvement of the thermosensitive calcium channel TRPV4. The study presents interesting observations and could be relevant to understanding host determinants of coronavirus replication. However, the mechanistic link between TRPV4 and temperature-dependent viral replication remains insufficiently defined, and the conclusions would benefit from further clarification and additional validation. Several major issues remain that weaken the strength of the conclusions:

Major points:

1. The main conclusion that TRPV4 plays a predominant role in temperature-dependent SARS-CoV-2 replication appears overstated. In Fig. 2G-J, TRPV4 knockdown reduced Omicron BA.5 gRNA levels by ~10-fold, whereas culturing at 34 °C reduced gRNA levels by ~400-fold (Fig. 1C). While TRPV4 knockdown reduces viral replication moderately (Fig. 2H–J), lowering culture temperature results in a much more profound effect (Fig. 1C). This discrepancy suggests that other factors, such as direct effects of temperature on viral enzymatic activity (e.g., RdRp) or RNA stability, are likely to play dominant roles and should be considered more carefully.

   We thank the reviewer for this important comment regarding the relative contribution of viral enzymatic activity versus host factors in temperature-dependent SARS-CoV-2 replication.

   We agree that temperature may directly influence viral processes such as RNA-dependent RNA polymerase (RdRp) activity. To address this possibility, we experimentally assessed RdRp activity using a commercially available SARS-CoV-2 RdRp assay kit. Interestingly, we found that RdRp activity was not highest at 37°C but instead peaked at 34°C under our experimental conditions (new Fig. S3). This observation is notable because the assay protocol is optimized for incubation at 37°C, yet higher enzymatic activity was detected at a lower temperature. These results suggest that intrinsic viral polymerase activity is not enhanced at 37°C and therefore does not explain the increased viral replication observed at this temperature. Importantly, despite higher RdRp activity at 34°C, viral replication in cells was markedly reduced compared to 37°C (Fig. 1, Fig. S1 and S2). This discrepancy indicates that viral enzymatic activity alone is insufficient to account for the temperature-dependent replication efficiency of SARS-CoV-2. Taken together, our findings support the notion that host factors, rather than direct effects of temperature on viral enzymes, play a dominant role in promoting efficient viral replication at 37°C.

In this context, our data demonstrate that TRPV4-mediated calcium influx is one such critical host mechanism contributing to this phenomenon. To reflect this more balanced interpretation, we have revised the relevant text in the manuscript to clarify that TRPV4 is a key contributing factor, while acknowledging that multiple mechanisms may be involved in temperature-dependent viral replication (lines 280-286 page 14).

2. The mechanistic connection between SARS-CoV-2 infection and TRPV4 activation remains unclear. Although infection elevates intracellular Ca^2 levels (Fig. S5), it is not demonstrated whether this is specifically mediated by TRPV4. Direct comparison of Ca^2 signals under TRPV4 inhibition or knockdown would help clarify this point.

   We thank the reviewer for this insightful comment regarding the mechanistic link between SARS-CoV-2 infection and TRPV4-mediated Ca^{2+} influx. To directly address this point, we performed additional experiments using TRPV4 knockdown cells to determine whether SARS-CoV-2-induced Ca^{2+} influx is mediated by TRPV4. As shown in the newly added Supplementary Figure (Fig. S11), SARS-CoV-2 infection induced a significant increase in intracellular Ca^{2+} levels in control cells, whereas this increase was markedly attenuated in TRPV4 knockdown cells. These results demonstrate that SARS-CoV-2-induced Ca^{2+} influx is largely dependent on TRPV4, thereby providing direct mechanistic evidence linking viral infection to TRPV4 activation. Together with our previous findings showing that pharmacological activation and inhibition of TRPV4 modulate Ca^{2+} influx (Fig. S7 and S8) and viral replication (Fig. 2), these data strengthen our conclusion that TRPV4-mediated calcium entry plays a critical role in SARS-CoV-2 infection. We have incorporated these new data into the revised manuscript and updated the Results and Discussion sections accordingly (lines 179-181 page 9).

3. The pathway by which SARS-CoV-2 activates TRPV4 is not addressed. At present, the mechanism remains speculative, and additional discussion or experimental support would be needed to substantiate this link.

   We thank the reviewer for this important comment regarding the mechanism by which SARS-CoV-2 activates TRPV4. We agree that the precise upstream pathway linking viral infection to TRPV4 activation remains to be fully elucidated. To gain further insight into this process, we performed additional experiments using heat-inactivated SARS-CoV-2. As shown in the newly added Supplementary Figure (Fig. S10), infection with live SARS-CoV-2 induced a robust increase in intracellular Ca^{2+}

levels, whereas heat-inactivated virus failed to do so. These results indicate that TRPV4 activation is not triggered merely by viral particle binding or entry, but rather requires active viral processes associated with replication. Thus, while the precise molecular pathway remains to be determined, our data provide experimental evidence that TRPV4 activation depends on biologically active virus. We have now included these findings in the revised manuscript and expanded the Discussion to more clearly acknowledge the remaining mechanistic questions (lines 177-179 page 9, lines 300-303 page 15).

4. The exclusive focus on TRPV4 does not rule out potential contributions from other thermosensitive Ca^2 channels, which should be discussed as possible alternatives or complementary pathways.

   We thank the reviewer for raising this important point regarding the potential contribution of other thermosensitive Ca^{2+} channels. We agree that other channels, such as TRPV1 or TRPV3, may also contribute to calcium signaling under certain conditions. However, to examine whether the requirement for TRPV4 is context-dependent, we generated a cold-adapted SARS-CoV-2 Omicron BA.5 variant that efficiently replicates at lower temperatures. As shown in the newly added Supplementary Figure (Fig. S6), the cold-adapted virus exhibited enhanced replication at 21°C compared to the parental virus. Notably, under these low-temperature conditions, knockdown of TRPV4 did not affect viral replication, in contrast to the clear TRPV4 dependence observed at 37°C. These findings indicate that TRPV4 is not universally required for SARS-CoV-2 replication, but rather plays a temperature-dependent role under physiological conditions. Therefore, while other Ca^{2+} channels may contribute under specific circumstances, our data demonstrate that TRPV4 is a key determinant of efficient viral replication at core body temperature. We have now included these results in the revised manuscript and expanded the Discussion to clarify this point (lines 160-168 page 9, lines 324-327 page 16).

Minor Comments

1. In Fig. 1, WT, Delta, and Omicron BA.1 replicate best at 37 °C, with reduced replication at 34 °C and 39 °C. Omicron BA.5 appears most temperature-sensitive, yet it was not included in later validation experiments. The rationale should be clarified.

   We thank the reviewer for this helpful comment. We selected representative SARS-CoV-2 variants for detailed mechanistic analyses to balance experimental feasibility

with the ability to capture conserved biological features. In particular, we used the Delta variant for most mechanistic experiments, as it showed robust and reproducible replication across temperature conditions, making it well-suited for functional studies. Importantly, key findings were also confirmed using the Omicron BA.5 variant (Fig. 2I and related data), indicating that the observed TRPV4-dependent effects are not restricted to a single viral strain. Although BA.5 exhibited relatively higher temperature sensitivity in Fig. 1, the overall temperature-dependent replication pattern was consistent across variants. In addition, the use of the Delta variant enabled direct alignment with our in vivo hamster experiments. Notably, in our experimental system, lethal infection in Syrian hamsters was reliably achieved using the Delta variant, whereas other variants, including Omicron, did not consistently induce severe disease. Therefore, the Delta variant was selected for the in vivo studies to ensure a robust and physiologically relevant disease model.

Taken together, we believe that the selected experimental design is sufficient to support the generality of our conclusions. We have now clarified this rationale in the revised manuscript (lines 114-116 page 7).

2. Issues related to Fig. 2:

a. The rationale for testing TRPV4 directly, without first screening other candidate channels (e.g., TRPV3, TRPV8), should be explained.

We thank the reviewer for this thoughtful comment. We selected TRPV4 as a primary candidate based on its well-established thermosensitivity within the physiological temperature range (~37°C) and its known role in mediating calcium influx under these conditions. In particular, TRPV4 is activated near core body temperature, making it a strong candidate for linking temperature to SARS-CoV-2 replication efficiency. Our study was therefore designed to test a hypothesis-driven mechanism focusing on TRPV4, rather than performing a broad screening of multiple thermosensitive channels. Importantly, we provide multiple independent lines of evidence supporting the role of TRPV4, including pharmacological modulation, genetic knockdown, and calcium influx measurements. In addition, as suggested by the reviewer, we have now expanded the Discussion and included new experimental data (Fig. S6) demonstrating that TRPV4 dependency is temperature-dependent, supporting the idea that alternative pathways may contribute under different conditions. We have clarified this rationale in the revised manuscript (lines 133-136 pages 7-8).

b. In Fig. 2D-E, drug treatment (5 μM and 50 μM) reduced gRNA more strongly than 34 °C incubation. Please provide Delta infection results at 34 °C (gRNA and titers) from the same batch for comparison.

We thank the reviewer for this helpful suggestion. The experiments shown in Fig. 2D–E (drug treatment) and Fig. 1C (temperature-dependent replication) were performed independently and were not designed for direct quantitative comparison. Therefore, differences in the magnitude of inhibition between these conditions should be interpreted with caution. Importantly, the purpose of Fig. 2D–E was to demonstrate that pharmacological inhibition of TRPV4 suppresses viral replication under physiological temperature conditions, rather than to directly compare the extent of inhibition with that observed at lower temperatures. Nevertheless, both sets of data consistently support the conclusion that SARS-CoV-2 replication is sensitive to both temperature changes and TRPV4 activity. We have clarified this point in the revised manuscript to avoid potential misinterpretation (lines 151-153 page 8).

c. In Fig. 2G-J, the possibility of off-target effects of TRPV4 knockdown cannot be excluded. A rescue experiment restoring TRPV4 expression should be performed to confirm specificity.

We thank the reviewer for this important comment regarding the specificity of TRPV4 knockdown. We agree that off-target effects are an important consideration in knockdown-based approaches. However, in our study, we provide multiple independent lines of evidence supporting the specificity of TRPV4 involvement. First, TRPV4 knockdown selectively impaired Ca^{2+} influx induced by the TRPV4 agonist GSK1016790A, whereas ATP-induced Ca^{2+} influx remained unaffected (Fig. S8), indicating that general calcium signaling pathways are preserved. Second, pharmacological modulation of TRPV4 using both an agonist and an antagonist consistently affected viral replication (Fig. 2A–F), supporting a specific role for TRPV4. Third, SARS-CoV-2-induced Ca^{2+} influx was significantly reduced in TRPV4 knockdown cells (Fig. S11), further linking TRPV4 to virus-induced calcium signaling. Taken together, these orthogonal approaches strongly support the specificity of TRPV4 involvement and make it unlikely that the observed effects are due to off-target knockdown effects. While a rescue experiment could further strengthen specificity, such experiments are technically challenging in this system and were beyond the scope of the present study. We have now clarified this point in the revised manuscript (lines 327-329 page 16).

3. Fig. 3 highlights the role of Ca2+ influx by showing that ionomycin and BAPTA-AM modulate infection. However, since both compounds act broadly on intracellular Ca2+, it remains difficult to attribute the effect specifically to TRPV4.

   We thank the reviewer for this important comment. We agree that ionomycin and BAPTA-AM broadly modulate intracellular Ca^{2+} levels and are not specific to TRPV4. Therefore, the purpose of these experiments was not to demonstrate TRPV4 specificity, but rather to assess the general contribution of Ca^{2+} influx to SARS-CoV-2 replication. Importantly, the specificity of TRPV4 involvement is supported by independent lines of evidence, including pharmacological modulation using TRPV4-specific agonists and antagonists (Fig. 2), genetic knockdown of TRPV4 (Fig. 2G–J), and selective impairment of TRPV4-dependent Ca^{2+} influx (Fig. S11).

   Taken together, these results indicate that while Ca^{2+} influx in general promotes viral replication, TRPV4 represents a key upstream regulator of this process under physiological temperature conditions. We have clarified this point in the revised manuscript (lines 194-196 page 10).

4. Fig. 4 demonstrates that calcineurin inhibitors reduce infection in cells and hamsters. While this supports a role for Ca2+-calcineurin signaling, the direct mechanistic link to TRPV4 is missing. The authors should clarify how calcineurin promotes SARS-CoV-2 infection.

   We thank the reviewer for this insightful comment. We agree that the precise molecular mechanisms by which calcineurin promotes SARS-CoV-2 infection remain to be fully elucidated. However, detailed dissection of downstream signaling pathways was beyond the scope of the present study. In this work, our aim was to identify upstream host factors that link temperature-dependent calcium influx to viral replication. Our data consistently indicate that TRPV4-mediated Ca^{2+} influx promotes SARS-CoV-2 replication, and that inhibition of downstream Ca^{2+}-dependent signaling, including calcineurin, suppresses viral infection both in vitro and in vivo. Thus, we interpret calcineurin as a downstream effector of TRPV4-mediated calcium signaling, rather than as an independent upstream regulator. While the precise molecular mechanisms by which calcineurin enhances viral replication warrant further investigation, our findings establish a functional TRPV4-Ca^{2+}-calcineurin axis that supports SARS-CoV-2 infection. We have clarified this point in the revised Discussion (lines 315-317 page 15).

Part I - Summary

Please use this section to discuss strengths/weaknesses of study, novelty/significance, general execution and scholarship.

Reviewer #2: Kobayashi et al in the manuscript "TRPV4-mediated calcium influx governs temperature-dependent SARS-CoV-2 replication" identify TRPV4-mediated calcium influx as a mechanism promoting optimal SARS-CoV-2 replication at 37°C and show that the calcium channel blocker mandipine reduces viral load and improves survival in hamsters. While this offers a novel mechanistic insight into temperature-dependent viral replication, the conclusions are constrained by the choice of model systems and modest phenotypic effects. Below are key points for the authors' consideration:

Major:

1. The research relies heavily on VeroE6/TMPRSS2 cells, a monkey kidney cell line that is deficient in the type-I interferon response, a critical arm of the innate immune system; this limits the ability to generalize the findings to more physiologically relevant human respiratory cells and to understand the role of immunity in this temperature-sensitive process.

We thank the reviewer for raising this important point regarding the reliance on VeroE6/TMPRSS2 cells, which are deficient in type I interferon responses. To address this concern, we have now performed additional experiments using human respiratory cell models, including Calu-3 cells and primary Human Tracheal Epithelial Cells (HTEpC), both of which possess intact innate immune signaling pathways, including type I interferon responses. In these physiologically relevant human cells, we observed temperature-dependent differences in SARS-CoV-2 replication that were highly consistent with those obtained in VeroE6/TMPRSS2 cells. Specifically, viral RNA levels showed the same temperature-dependent trends across 34°C, 37°C, and 39°C conditions. Importantly, we extended these analyses to include time-course experiments, demonstrating that this temperature-dependent phenotype is maintained dynamically over the course of infection in both Calu-3 and HTEpC cells (new Fig. S2).

These results indicate that the observed temperature sensitivity of SARS-CoV-2 replication is not an artifact of the interferon-deficient VeroE6/TMPRSS2 system, but rather represents a robust and reproducible phenomenon that is conserved across multiple cell types, including human airway epithelial cells with intact innate immune responses. Taken together, our findings strengthen the physiological relevance of our

conclusions and suggest that the temperature-dependent regulation of SARS-CoV-2 replication is largely independent of cell-type-specific differences in interferon competency. We have clarified this point in the revised manuscript (lines 103-114 pages 6-7, lines 258-261 page 13).

2. The observed phenotypes are relatively mild. For instance, TRPV4 knockdown only reduced viral gRNA by less than 1-log, suggesting it may not be the primary mediator of temperature-dependent calcium flux. The TRPV family includes multiple members (e.g., TRPV1-6); further justification is needed for the specific focus on TRPV4 as the key player.

We thank the reviewer for this important comment. We agree that the magnitude of reduction in viral gRNA upon TRPV4 knockdown is modest (<1 log), and we do not claim that TRPV4 acts as a primary driver of SARS-CoV-2 replication. Rather, we propose that TRPV4 functions as a temperature-sensitive facilitator that contributes to efficient viral replication under physiological temperature conditions.

Viral replication is a multifactorial process regulated by both viral and host determinants, and it is common that individual host factors contribute modestly but significantly to overall replication efficiency. In this context, the observed reduction is consistent with a modulatory, rather than dominant, role of TRPV4.

Regarding the specificity of TRPV4 among TRPV family members, our focus on TRPV4 is based on its well-characterized activation within the physiological temperature range and its established role in mediating Ca^{2+} influx in epithelial cells. In addition, our data demonstrate that TRPV4-dependent Ca^{2+} influx is selectively impaired in knockdown cells, while ATP-induced Ca^{2+} responses are preserved (Fig. S8), supporting the specificity of this pathway.

Importantly, our new experiments using a cold-adapted SARS-CoV-2 variant further support the context-dependent role of TRPV4. At low temperature (21°C), replication of the cold-adapted virus was not affected by TRPV4 knockdown (Fig. S6), in contrast to the parental virus at 37°C. These findings indicate that TRPV4 is not a general requirement for viral replication but specifically contributes to temperature-dependent viral propagation.

Taken together, we propose that TRPV4 represents a key host factor that modulates temperature-dependent SARS-CoV-2 replication, rather than a universal regulator of viral replication. We have clarified this rationale in the revised manuscript (lines 133-136 pages 7-8).

3. The therapeutic data for mandipine are preliminary. Crucial experiments are missing to demonstrate that its antiviral effect is specifically due to TRPV4 inhibition and not its known off-target activity against the viral main protease (Mpro).

We thank the reviewer for this important comment regarding the mechanism underlying the antiviral effect of manidipine. We agree that manidipine has been reported to inhibit the SARS-CoV-2 main protease (Mpro), and therefore its antiviral activity could potentially involve off-target effects beyond calcium channel inhibition. To address this concern, we performed additional in vivo experiments using a TRPV4-specific antagonist administered intravenously in SARS-CoV-2-infected hamsters.

As shown in the newly added Figure (Fig. 6), treatment with the TRPV4 antagonist significantly improved survival, reduced body weight loss, and decreased viral RNA levels and infectious titers compared to control animals. Importantly, this compound does not target viral Mpro, indicating that inhibition of TRPV4 signaling alone is sufficient to confer antiviral effects in vivo. These findings support the conclusion that host calcium signaling, specifically TRPV4-mediated pathways, plays a critical role in SARS-CoV-2 infection, and that the antiviral effect of manidipine is at least in part attributable to its modulation of host calcium influx rather than solely to direct viral enzyme inhibition.

We have incorporated these new data into the revised manuscript (lines 237-249 page 12) and clarified this point in the Discussion (lines 352-355 page 17).

4. The therapeutic data for mandipine are preliminary. Crucial experiments are missing to demonstrate that its antiviral effect is specifically due to TRPV4 inhibition and not its known off-target activity against the viral main protease (Mpro).

We thank the reviewer for this comment. This point overlaps with the concern raised in comment #3. As detailed in our response to comment #3, we have performed additional in vivo experiments using a TRPV4-specific antagonist (new Fig. 6), which demonstrate that inhibition of TRPV4 signaling alone is sufficient to confer antiviral effects, independent of direct viral Mpro inhibition. We respectfully refer the reviewer to our response to comment #3 for a detailed explanation.

5. The strong protective effect of intravenous cyclosporine A (Fig. 4K) is surprising, given its primary role as an immunosuppressant targeting T-cells. This seems inconsistent with the minimal reduction in viral load (Fig. 4I, J) and warrants further explanation.

We thank the reviewer for this insightful comment. We agree that the strong protective effect of cyclosporine A may appear unexpected given its well-known immunosuppressive activity and the relatively modest reduction in viral load. However,

increasing evidence indicates that disease severity in SARS-CoV-2 infection is not solely determined by viral burden, but also by dysregulated host inflammatory responses. In our previous study (Ref. #32; Kobayashi et al., PLoS Pathog, 2024), we demonstrated that excessive production of pro-inflammatory cytokines, including TNF-α, contributes to severe disease outcomes. Cyclosporine A is known to suppress calcineurin-dependent signaling pathways, thereby attenuating inflammatory responses. We therefore interpret the improved survival primarily as a consequence of reduced immunopathology rather than direct antiviral activity.

Regarding the reduction in viral load, although the effect is relatively modest, it is possible that inhibition of calcium-dependent host pathways may also partially impair viral replication. In addition, differences in the kinetics and anatomical distribution of viral replication versus inflammatory responses may contribute to the apparent discrepancy between viral load and survival outcomes. While the precise mechanisms by which cyclosporine A confers protection remain to be fully elucidated, our findings are consistent with a model in which modulation of host calcium–calcineurin signaling mitigates both viral replication and disease severity. We have clarified this point in the revised Discussion (lines 358-363 page 17).

6. The finding that TRPV4 effects are specific to 37°C and not observed at 34°C or 39°C (e.g., lines 111-112) is intriguing but requires more detailed mechanistic explanation.

We thank the reviewer for this insightful comment. We agree that the temperature specificity of TRPV4-dependent effects is an intriguing aspect of our findings. Although the precise mechanism underlying why these effects are most evident at 37°C remains to be fully elucidated, our current data support a model in which TRPV4 functions optimally within a narrow physiological temperature range close to core body temperature. Consistent with this interpretation, pharmacological activation of TRPV4 enhanced viral replication at 34°C, where endogenous TRPV4 activity is likely suboptimal, whereas this effect was not observed at 37°C or 39°C (Fig. S4). Conversely, pharmacological inhibition of TRPV4 suppressed viral replication at 37°C, but had minimal effects at 34°C or 39°C (Fig. S5). In addition, our data using a cold-adapted SARS-CoV-2 variant suggest that the contribution of TRPV4 is context-dependent and may be bypassed under non-physiological low-temperature conditions (Fig. S6).

Taken together, these findings suggest that TRPV4 contributes to SARS-CoV-2 replication most effectively under physiological temperature conditions, while other host or viral factors may become limiting or compensatory outside this range. We have clarified this point in the revised Discussion (lines 317-321 page 15).

7. The potential effect of temperature on other essential factors for viral replication (e.g., receptor expression, viral entry, or replication complex activity) is not adequately discussed and should be addressed.

We thank the reviewer for this important comment. We agree that temperature may influence multiple aspects of the viral life cycle, including receptor expression, viral entry, and replication complex activity. In the present study, our primary objective was to identify host factors that link physiological temperature to enhanced SARS-CoV-2 replication, and we focused on TRPV4-mediated calcium signaling as a key mechanism. Importantly, our data suggest that intrinsic viral enzymatic activity alone does not account for the temperature-dependent replication, as RdRp activity was highest at 34°C despite reduced viral replication in cells (Fig. S3). This finding supports the notion that host-dependent mechanisms play a dominant role under physiological temperature conditions.

We acknowledge that additional factors, such as temperature-dependent changes in receptor expression, membrane dynamics, or viral replication complex assembly, may also contribute to viral replication efficiency. These possibilities represent important areas for future investigation. We have now expanded the Discussion to address these points and to more clearly position our findings within the broader context of temperature-dependent viral replication (lines 329-336 page 16).

Minor:

1. The first sentence of the abstract is cumbersome and should be rephrased for clarity: "While there is considerable variation in the temperatures of the nasal cavity, lower respiratory tract, and feverish states, the impact of these variations on SARS-CoV-2 replication remains less clear."

We thank the reviewer for this helpful suggestion. We agree that the first sentence of the abstract could be improved for clarity. We have revised the sentence accordingly in the manuscript (lines 19-21 page 2).

2. A full replication kinetics curve, rather than single time-point titers (e.g., Figure 1), would more accurately depict the dynamics of viral replication.

We thank the reviewer for this helpful suggestion. In response, we have performed additional experiments to assess the full replication kinetics of SARS-CoV-2 at different temperatures. As shown in the newly added Figure (Fig. S1 and S2), we measured viral genomic RNA levels at multiple time points post-infection for ancestral, Delta, and Omicron BA.5 variants. These data clearly demonstrate that SARS-CoV-2 replication is

most efficient at 37°C across all tested variants, while replication is reduced at both 34°C and 39°C throughout the time course. The kinetics further confirm and extend our original observations by providing a more comprehensive view of temperature-dependent viral replication dynamics. We have incorporated these new data into the revised manuscript and updated the Results section accordingly (lines 98-110 pages 6-7).

3. There is a substantial discrepancy in the baseline Delta variant gRNA levels between figures (e.g., >10^5 in Fig. 1B vs. 10^2 in Fig. 2A), which should be clarified.

We thank the reviewer for this helpful comment. The apparent discrepancy in baseline gRNA levels across figures arises from the normalization strategy used in our qRT-PCR analyses. Specifically, viral genomic RNA (gRNA) levels were normalized to the value obtained from mock-infected supernatants, which was set to 1 in each independent experiment. Because the baseline signal in mock controls can vary between experiments, the absolute scale of the normalized values is not directly comparable across figures. Importantly, all comparisons within each figure were performed using samples processed in the same experiment, and therefore the relative differences shown are valid. We have clarified this point in the Methods and figure legends to avoid confusion (lines 455-457 page 22, lines 771-773 page 33, and lines 805-807 page 35).

Part II – Major Issues: Key Experiments Required for Acceptance

Please use this section to detail the key new experiments or modifications of existing experiments that should be absolutely required to validate study conclusions.

Generally, there should be no more than 3 such required experiments or major modifications for a "Major Revision" recommendation. If more than 3 experiments are necessary to validate the study conclusions, then you are encouraged to recommend "Reject".

Reviewer #2: See the review summary.

Part III – Minor Issues: Editorial and Data Presentation Modifications

Please use this section for editorial suggestions as well as relatively minor modifications of existing data that would enhance clarity.
