# Supplementary material for: TRPV4-mediated calcium influx contributes temperature-dependent SARS-CoV-2 replication
Source: PLoS Pathog. 2026 Aug 21;22(8):e1014520. doi: 10.1371/journal.ppat.1014520 (PMC13524343; doi:10.1371/journal.ppat.1014520)
Supplement: Peer review history

# Author comment: Author response to Decision Letter 0

**DOI:** 10.1371/journal.ppat.1014520.r001

27 Apr 2026

Attachment Submitted filename: Reviewer comments 260426 v2.pdf

# Peer review: Decision Letter 0

**DOI:** 10.1371/journal.ppat.1014520.r002

**Authors:** Ashley L. St. John, Yaw Shin Ooi

7 Jul 2026

TRPV4-mediated calcium influx governs temperature-dependent SARS-CoV-2 replication

PLOS Pathogens

Dear Dr. Ichinohe,

Thank you for submitting your manuscript to PLOS Pathogens. After careful consideration, we feel that it has merit but does not fully meet PLOS Pathogens's publication criteria as it currently stands. Therefore, we invite you to submit a revised version of the manuscript that addresses the points raised during the review process.

Please submit your revised manuscript by Sep 05 2026 11:59PM. If you will need more time than this to complete your revisions, please reply to this message or contact the journal office at plospathogens@plos.org. When you're ready to submit your revision, log on to https://www.editorialmanager.com/ppathogens/ and select the 'Submissions Needing Revision' folder to locate your manuscript file.

Please include the following items when submitting your revised manuscript:

* A letter that responds to each point raised by the editor and reviewer(s). You should upload this letter as a separate file labeled 'Response to Reviewers'. This file does not need to include responses to any formatting updates and technical items listed in the 'Journal Requirements' section below.

* A marked-up copy of your manuscript that highlights changes made to the original version. You should upload this as a separate file labeled 'Revised Manuscript with Track Changes'.

* An unmarked version of your revised paper without tracked changes. You should upload this as a separate file labeled 'Manuscript'.

We look forward to receiving your revised manuscript.

Kind regards,

Yaw Shin Ooi, PhD

Academic Editor

PLOS Pathogens

Ashley St. John

Section Editor

Editor-in-Chief

PLOS Pathogens

orcid.org/0000-0003-2946-9497

Editor-in-Chief

PLOS Pathogens

orcid.org/0000-0002-7699-2064

**Additional Editor Comments (if provided):**

Thank you for submitting the revised version of your manuscript. The revised manuscript has now been evaluated by the reviewers.

The reviewers acknowledge that the revision has addressed many of the concerns raised previously. In particular, the evidence supporting a role for intracellular calcium in SARS-CoV-2 replication and TRPV4 as a contributor to this process is considered convincing. However, concerns remain regarding the central mechanistic conclusion that the TRPV4–Ca²⁺ axis underlies temperature-dependent SARS-CoV-2 replication.

In preparing your revision, please carefully consider whether the overall framing and conclusions of the manuscript are fully supported by the experimental evidence. If the current data do not directly establish the mechanistic link between temperature and the TRPV4–Ca²⁺ axis, the title, abstract, Results, and Discussion should be revised accordingly to ensure that the conclusions accurately reflect the scope of the data. Please also address all reviewer comments in a detailed point-by-point response.

**Journal Requirements:**

If the reviewer comments include a recommendation to cite specific previously published works, please review and evaluate these publications to determine whether they are relevant and should be cited. There is no requirement to cite these works unless the editor has indicated otherwise.

1) Your Response to Reviewers should be uploaded as a single, individual file. Please remove your Response to Reviewers from your Cover Letter.

2) We have noticed that you have uploaded Supporting Information files, but you have not included a list of legends. Please add a full list of legends for your Supporting Information files after the references list.

3) Please amend your detailed Financial Disclosure statement. This is published with the article. It must therefore be completed in full sentences and contain the exact wording you wish to be published.

i) State what role the funders took in the study. If the funders had no role in your study, please state: "The funders had no role in study design, data collection and analysis, decision to publish, or preparation of the manuscript."

ii) If any authors received a salary from any of your funders, please state which authors and which funders..

If you did not receive any funding for this study, please simply state: u201cThe authors received no specific funding for this work.u201d

4) PLOS now requires that authors provide the original uncropped and unadjusted images underlying all blot or gel results reported in a submission’s figures or Supporting Information files. This policy and the journal’s other requirements for blot/gel reporting and figure preparation are described in detail at https://journals.plos.org/plosone/s/figures#loc-blot-and-gel-reporting-requirements and https://journals.plos.org/plosone/s/figures#loc-preparing-figures-from-image-files.

When you submit your revised manuscript, please ensure that your figures adhere fully to these guidelines and provide the original underlying images for all blot or gel data reported in your submission. See the following link for instructions on providing the original image data: https://journals.plos.org/plosone/s/figures#loc-original-images-for-blots-and-gels.

In your cover letter, please note whether your blot/gel image data are in Supporting Information or posted at a public data repository, provide the repository URL if relevant, and provide specific details as to which raw blot/gel images, if any, are not available. Email us at customercare@plos.org if you have any questions.

**Reviewers' Comments:**

Reviewer's Responses to Questions

**Part I - Summary**

Please use this section to discuss strengths/weaknesses of study, novelty/significance, general execution and scholarship.

Reviewer #1: I sincerely thank the authors for thoroughly addressing all of my concerns.

Reviewer #2: Kobayashi and Abe et al. identify TRPV4, a temperature-dependent calcium channel, as a major contributor to the temperature-dependent replication of SARS-CoV-2 at physiologically relevant temperatures: 34°C, representing the upper respiratory tract; 38°C, representing core body temperature; and 39°C, representing a fever-like condition. The authors show consistent trends across three viral variants. Their mechanistic evidence is supported by a comprehensive set of experiments focused on calcium influx. Specifically, they use a TRPV4 agonist at 34°C to increase viral replication and a TRPV4 antagonist at 37°C to inhibit replication. To further support calcium influx as a key factor in temperature-sensitive SARS-CoV-2 replication, they use the broad calcium ionophore ionomycin and the calcium chelator BAPTA-AM to manipulate intracellular calcium levels, producing results consistent with the TRPV4 experiments. The controls are thorough and well designed, including assessments of drug toxicity, potential off-target effects on proteins known to affect SARS-CoV-2 infection such as ACE2 and TMPRSS2, and treatment effects at the relevant temperatures. In addition, the authors perform genetic siRNA knockdown experiments and show that SARS-CoV-2 replication is reduced following TRPV4 knockdown at 37°C. The authors also perform in vivo experiments in a hamster model. Treatment with the general calcium channel blockers cyclosporin A and manidipine has a significant effect on survival. In contrast, a TRPV4-specific inhibitor shows only a modest protective effect in animals. Overall, the data are well controlled and carefully executed, with substantial experimental replication.

Reviewer #3: This manuscript identify the TRPV4 play an important role during the SARS-CoV-2 infection cycle by regulation calcium concentration as the thermosensitive channel. Meanwhile, the authors tested the therapeutic potential of targeting calcium channel in hamster. However, the mechanistic interaction between the TRPV4 and SARS-CoV-2 need further clarification and validation. There have been one review about the host cell calcium dynamics and SARS-CoV-2 infection (https://doi.org/10.1016/j.ceca.2022.102637). Several issues should be further explored or clarification.

**********

**Part II – Major Issues: Key Experiments Required for Acceptance**

Please use this section to detail the key new experiments or modifications of existing experiments that should be absolutely required to validate study conclusions.

Generally, there should be no more than 3 such required experiments or major modifications for a "Major Revision" recommendation. If more than 3 experiments are necessary to validate the study conclusions, then you are encouraged to recommend "Reject".

Reviewer #1: No additional experiments required from my side.

Reviewer #2: n/a

Reviewer #3: The authors should be cautious with the quantification results of viral N gRNA, usually the RNA copies are hundreds or thousands times higher than the viral titers, but it is opposite in this study.

The authors investigated SARS-CoV-2 replication under different temperature (34 °C ,37°C , and 39 °C) ; however, the impact of temperature on cellular state was neither measured nor discussed, as most mammalian cell lines exhibit growth limitations at 39°C. Notably, it is worth noting that both TRPV4 agonists and inhibitors had minimal effects on SARS-CoV-2 replication.

It is interesting to sequence the genome of the cold-adapted SARS-CoV-2, then check the sequence mutations of the cold-adapted variants and confirm the locations of the mutations. This may help us identify which proteins play important functions during the temperature-dependent process. The authors should discuss this point in the manuscript.

In the Figure 2E and 3F, the virus titer value of PFU for the control group looks same, are they independent experiments or use the same control?

**********

**Part III – Minor Issues: Editorial and Data Presentation Modifications**

Please use this section for editorial suggestions as well as relatively minor modifications of existing data that would enhance clarity.

Reviewer #1: (No Response)

Reviewer #2: The calcineurin-related data are less clear. Although these experiments appear to be well executed, they do not currently add substantial mechanistic insight. If the authors intend to link calcium influx to downstream calcineurin signaling as an in-depth mechanism, additional experiments will be needed to support this connection more directly. In its current form, this section introduces some confusion rather than strengthening the overall manuscript.

Although the evidence that calcium influx affects SARS-CoV-2 replication is convincing, and the role of TRPV4 appears significant, the authors should be cautious in claiming that temperature-dependent SARS-CoV-2 replication is driven specifically by TRPV4. The data support TRPV4 as an important contributor, but none of the experiments demonstrate complete rescue to the level observed at 37°C. Furthermore, the in vivo experiment of TRPV4 inhibitor is not convincing. The survival curves have very minor differences between the mock and treatment groups. Why are there only days 3 and 5 weight loss shown? Why was only day 3 titer significant?

Overall, the manuscript is well done and just requires some changes in wording to avoid over claiming the significant. For instance: in the title, “governs” may be too strong, “influence” or similar terms may be more appropriate.

Reviewer #3: (No Response)

**********

PLOS authors have the option to publish the peer review history of their article (what does this mean?). If published, this will include your full peer review and any attached files.

If you choose “no”, your identity will remain anonymous but your review may still be made public.

**Do you want your identity to be public for this peer review?**  For information about this choice, including consent withdrawal, please see our Privacy Policy.

Reviewer #1: No

Reviewer #2: **Yes:**  Long Ping Victor Tse

Reviewer #3: No

[NOTE: If reviewer comments were submitted as an attachment file, they will be attached to this email and accessible via the submission site. Please log into your account, locate the manuscript record, and check for the action link "View Attachments". If this link does not appear, there are no attachment files.]

**Figure resubmission:**

**Reproducibility:**

?>

# Author comment: Author response to Decision Letter 1

**DOI:** 10.1371/journal.ppat.1014520.r003

16 Jul 2026

Attachment Submitted filename: Response to Reviewers 260714.pdf

# Peer review: Decision Letter 1

**DOI:** 10.1371/journal.ppat.1014520.r004

**Authors:** Ashley L. St. John, Yaw Shin Ooi

4 Aug 2026

Dear Dr. Ichinohe,

We are pleased to inform you that your manuscript 'TRPV4-mediated calcium influx contributes temperature-dependent SARS-CoV-2 replication' has been provisionally accepted for publication in PLOS Pathogens.

Before your manuscript can be formally accepted you will need to complete some formatting changes, which you will receive in a follow up email. A member of our team will be in touch with a set of requests.

Please note that your manuscript will not be scheduled for publication until you have made the required changes, so a swift response is appreciated.

IMPORTANT: The editorial review process is now complete. PLOS will only permit corrections to spelling, formatting or significant scientific errors from this point onwards. Requests for major changes, or any which affect the scientific understanding of your work, will cause delays to the publication date of your manuscript.

Should you, your institution's press office or the journal office choose to press release your paper, you will automatically be opted out of early publication. We ask that you notify us now if you or your institution is planning to press release the article. All press must be co-ordinated with PLOS.

Thank you again for supporting Open Access publishing; we are looking forward to publishing your work in PLOS Pathogens.

Best regards,

Yaw Shin Ooi, PhD

Academic Editor

PLOS Pathogens

Ashley St. John

Section Editor

PLOS Pathogens

Sumita Bhaduri-McIntosh

Editor-in-Chief

PLOS Pathogens

orcid.org/0000-0003-2946-9497

Michael Malim

Editor-in-Chief

PLOS Pathogens

orcid.org/0000-0002-7699-2064

***********************************************************

Reviewer Comments (if any, and for reference):

Reviewer's Responses to Questions

**Part I - Summary**

Please use this section to discuss strengths/weaknesses of study, novelty/significance, general execution and scholarship.

Reviewer #2: I have no additional comment.

Reviewer #3: The authors have answered the questions properly.

**********

**Part II – Major Issues: Key Experiments Required for Acceptance**

Please use this section to detail the key new experiments or modifications of existing experiments that should be absolutely required to validate study conclusions.

Generally, there should be no more than 3 such required experiments or major modifications for a "Major Revision" recommendation. If more than 3 experiments are necessary to validate the study conclusions, then you are encouraged to recommend "Reject".

Reviewer #2: (No Response)

Reviewer #3: The authors have answered the questions properly.

**********

**Part III – Minor Issues: Editorial and Data Presentation Modifications**

Please use this section for editorial suggestions as well as relatively minor modifications of existing data that would enhance clarity.

Reviewer #2: (No Response)

Reviewer #3: No

**********

PLOS authors have the option to publish the peer review history of their article (what does this mean?). If published, this will include your full peer review and any attached files.

If you choose “no”, your identity will remain anonymous but your review may still be made public.

**Do you want your identity to be public for this peer review?**  For information about this choice, including consent withdrawal, please see our Privacy Policy.

Reviewer #2: **Yes:**  Long Ping Victor Tse

Reviewer #3: No

# Editor report: Acceptance letter

**DOI:** 10.1371/journal.ppat.1014520.r005

**Authors:** Ashley L. St. John, Yaw Shin Ooi

Dear Dr. Ichinohe,

We are delighted to inform you that your manuscript, "TRPV4-mediated calcium influx contributes temperature-dependent SARS-CoV-2 replication," has been formally accepted for publication in PLOS Pathogens.

We have now passed your article onto the PLOS Production Department who will complete the rest of the pre-publication process. All authors will receive a confirmation email upon publication.

The corresponding author will soon be receiving a typeset proof for review, to ensure errors have not been introduced during production. Please review the PDF proof of your manuscript carefully, as this is the last chance to correct any scientific or type-setting errors. Please note that major changes, or those which affect the scientific understanding of the work, will likely cause delays to the publication date of your manuscript. Note: Proofs for Front Matter articles (Pearls, Reviews, Opinions, etc...) are generated on a different schedule and may not be made available as quickly.

Soon after your final files are uploaded, the early version of your manuscript, if you opted to have an early version of your article, will be published online. The date of the early version will be your article's publication date. The final article will be published to the same URL, and all versions of the paper will be accessible to readers.

For Research Articles, you will receive an invoice from PLOS for your publication fee after your manuscript has reached the completed accept phase. If you receive an email requesting payment before acceptance or for any other service, this may be a phishing scheme. Learn how to identify phishing emails and protect your accounts at https://explore.plos.org/phishing.

Thank you again for supporting open-access publishing; we are looking forward to publishing your work in PLOS Pathogens.

Best regards,

Sumita Bhaduri-McIntosh

Editor-in-Chief

PLOS Pathogens

orcid.org/0000-0003-2946-9497

Michael Malim

Editor-in-Chief

PLOS Pathogens

orcid.org/0000-0002-7699-2064
